# ApWD40a, a Member of the WD40-Repeat Protein Family, Is Crucial for Fungal Development, Toxin Synthesis, and Pathogenicity in the Ginseng Alternaria Leaf Blight Fungus *Alternaria panax*

**DOI:** 10.3390/jof11010059

**Published:** 2025-01-14

**Authors:** Jinling Lan, Shengjie Mei, Yingxue Du, Meili Chi, Jiayi Yang, Shuliu Guo, Mingliang Chu, Ronglin He, Jie Gao

**Affiliations:** 1 College of Plant Protection, Jilin Agricultural University, Changchun 130118, China; jinling_lan@aliyun.com (J.L.);; 2 National Ginseng Products Quality Inspection Testing Center, Yanji 133000, China

**Keywords:** *Alternaria panax*, WD40 tandemly repeated domains, secondary metabolite production, pathogenicity, transcriptome analysis, sulfate transport

## Abstract

*Alternaria panax*, the primary pathogen that causes ginseng Alternaria leaf blight disease, can lead to a 20–30% reduction in ginseng yield. WD40 repeat-containing proteins are evolutionarily conserved proteins with diverse functions between different organisms. In this study, we characterized the roles of a WD40 repeat-containing protein in *A. panax*. The deletion of *ApWD40a* impaired the mycelial growth, reduced the sporulation, and significantly decreased the efficiency in utilizing various carbon sources. The Δ*Apwd40a* mutant showed increased sensitivity to osmotic stress and metal ion stress induced by sorbitol, NaCl, and KCl, but decreased the sensitivity to a cell wall stress factor (SDS) and oxidative stress factors (paraquat and H_2_O_2_). Pathogenicity assays performed on detached ginseng leaves and roots revealed that the disruption of *ApWD40a* significantly decreased the fungal virulence through attenuating melanin and mycotoxin production by *A. panax*. A comparative transcriptome analysis revealed that *ApWD40a* was involved in many metabolic and biosynthetic processes, including amino acid metabolism, carbon metabolism, sulfate metabolic pathways, and secondary metabolite pathways. In particular, a significantly upregulated gene that encoded a sulfate permease 2 protein in Δ*Apwd40a*, named *ApSulP2*, was deleted in the wild-type strain of *A. panax*. The deletion of *ApSulP2* resulted in reduced biomass under sulfate-free conditions, demonstrating that the sulfate transport was impaired. Taken together, our findings highlight that *ApWD40a* played crucial roles in different biological processes and the pathogenicity of *A. panax* through modulating the expressions of genes involved in various primary and secondary metabolic processes.

## 1. Introduction

Ginseng (*Panax ginseng*) is a highly economically important plant that is extensively cultivated across East Asia, particularly northeastern China [1]. Long cultivation periods (generally 4–6 years) and strict requirements for the growth environment make ginseng susceptible to various diseases, such as grey mold, anthracnose, Alternaria leaf blight, and root rot [2,3,4,5]. Among these diseases, Alternaria leaf blight, caused by three species in the *Alternaria* genus (*Alternaria alternata*, *A. tenuissima*, and *A. panax*), can result in a 30% loss of ginseng yield, or even a 70% decrease in severe cases [6]. Of these three pathogens, *A. panax* specifically infects Araliaceae plants, including ginseng, American ginseng (*P. quinquefolius*), and Sanchi (*P. notoginseng*), and exhibits stronger pathogenicity than the other two species of *Alternaria*.

Previous studies of Alternaria leaf blight mainly focused on the isolation and identification of the pathogens, disease cycle, and disease control of ginseng using chemical pesticides and biocontrol agents, while little is known about the underlying pathogenic mechanisms of the fungal pathogens causing this blight [7,8]. Most recently, in 2024, small-molecule mycotoxins of *A. panax* were reported for the first time to be crucial for the pathogenicity of *A. panax* against Araliaceae plants [9]. Regarding the fungal pathogens of other major ginseng diseases, only two genes—*FoRSR1* in *Fusarium oxysporum* f. sp. *ginseng*, which causes root rot, and *BcSpd1* in *Botrytis cinerea*, which causes grey mold—were demonstrated to be involved in virulence and pathogenicity, both in 2023 [10,11]. There has been no reported study of the functional genes related to pathogenicity in *A. panax*. Investigating the pathogenic mechanisms of *A. panax* is thus of great significance for Alternaria leaf blight control.

The WD40 domain is one of the most abundant domains in eukaryotes. Eukaryotic organisms were revealed to possess a variety of WD40 structures, typically harboring 4–8 repeat units [12,13,14,15]. Despite their structural similarities, these proteins have evolved to serve distinct biological functions. The WD40 protein family was identified in a spectrum of organisms, including plants (*Arabidopsis thaliana*, tobacco (*Nicotiana tabacum*), rice (*Oryza sativa* L.), wheat (*Triticum aestivum* L.), and *Cucurbita maxima*) and fungi (*Saccharomyces cerevisiae, Aspergillus nidulans, Neurospora crassa, Magnaporthe oryzae, Metarhizium robertsii, and Ustilago maydis*) [16,17,18,19,20,21,22,23,24,25,26]. Research indicated that WD40-family proteins engage in transcriptional regulation and interact with a broad array of genes implicated in cell division, growth and development, carbon source utilization, biosynthesis of secondary metabolites, and abiotic stress responses [25,27]. Indeed, the WD40 protein family is pivotal for both plant and pathogenic fungal biology. In *Arabidopsis*, *SLOW WALKER1* (*SWA1*), which encodes a protein with six tandem WD40 repeats, is integral to 18S ribosomal RNA biogenesis. Silencing *SWA1* significantly impedes root growth due to the accumulation of unprocessed 18S precursor rRNA [28]. Similarly, in *N. tabacum*, it was reported that NtTTG2, containing the WD40 protein interaction domain, inhibits the nuclear localization of the protein NPR1 and the SA/NPR1-regulated defensive responses in plants, hence reducing the resistance to bacterial and viral infections [29]. Furthermore, in the pathogenic fungus *M. oryzae*, *MoCreC* and *MoTup1*, possessing five and seven WD40 repeat units, respectively, are essential for vegetative growth and pathogenicity. The Δ*MoCreC* and Δ*MoTup1* mutants display severe defects in these processes [24,30]. The Δ*MoCreC* mutant also exhibits a reduced mycelial growth rate on media with various carbon sources, which is a phenotype mirrored in CreC homologs in *A. nidulans* and *M. robertsii* [25,30,31]. In *U. maydis*, *RAK1*, containing seven tandem WD40 repeat units, is highly homologous to mammalian *RACK1* and the *S. cerevisiae* Asc1p [26,32]. The deletion of *RAK1* affects cell growth, cell wall integrity, and virulence-related cell fusion. Additionally, the Δ*rak1* mutant fails to respond to plant-derived stimuli, leading to attenuated pathogenicity [32,33]. *Tup1*, with seven tandem WD40 repeats, is crucial for mycelial growth, conidiation, macroconidial morphology, and pathogenicity in *F. oxysporum* and regulates the primary metabolic pathways [34].

In this study, we investigated the role of a gene containing six tandemly repeated WD40 domains within the WD40 protein family of *A. panax* using a targeted gene deletion strategy. We found that *ApWD40a* regulated the vegetative growth, stress responses, carbon source utilization, melanin and toxin production, and pathogenicity in *A. panax*. The RNA-seq analysis revealed that *ApWD40a* influenced the expression of genes that participated in the sulfur metabolism processes. Our results indicate that the upregulated gene *ApSulP2* in the mutant Δ*Apwd40a* was involved in sulfate transport but not in the pathogenicity of *A. panax*.

## 2. Materials and Methods

### 2.1. Fungal Strains, Growth Conditions, and Media

The *A. panax* strain, isolated from diseased ginseng leaves in Baishan, Jilin province, China, named JY15, served as the wild-type strain for all the experiments. The wild-type JY15, along with its derived variants, were grown on potato dextrose agar (PDA) in darkness at a temperature of 25 °C for routine cultivation. Sporulation analysis was conducted in V8 medium at 25 °C for 15 d. Induction medium was employed to induce the *Agrobacterium* culture, with the preparation method based on previously described procedures [35]. Minimal medium (MM), utilized for the phenotypic analysis, was composed of (per liter) 1% glucose, 0.6% NaNO_3_, 0.152% KH_2_PO_4_, 0.052% KCl, 0.052% MgSO_4_·7H_2_O, 0.0016% MnSO_4_·H_2_O, 0.00014% ZnSO_4_·7H_2_O, 0.0005% FeSO_4_·7H_2_O, 0.0002% CaCl_2_·2H_2_O, and 1.5% agar powder, with the pH adjusted to 5.5. The sulfate-free medium was formulated by replacing the sulfates in conventional MM with the corresponding chlorides. All fungal strains utilized in this study were preserved on filter paper blocks and stored at −20 °C for long-term conservation [5,6].

### 2.2. Disruption and Complementation of the ApWD40a Gene

Targeted gene replacement vectors were generated through homologous recombination using a ClonExpress Ultra One Step Cloning Kit (Vazyme Biotech Co., Ltd., Nanjin, China). Briefly, the 5′ (1311 bp) and 3′ (1269 bp) flanking sequences of the *ApWD40a* were amplified by PCR from the JY15 genome with specific primers (Table S1). The hygromycin (*HYG*) gene coding fragment was amplified by PCR from the pBS*-HPH* plasmid. The 5′ and 3′ flanking fragments, along with the *HYG* gene, were subjected to homologous recombination with the linearized pCAMBIA1300_modified (*Bam*H I/*Hin*d III) vector to construct the targeted replacement vectors. The vectors obtained were then directly transformed into *Agrobacterium tumefaciens* AGL-1, and co-transformation with protoplasts of JY15 was performed for gene knockout. Putative transformants were chosen on PDA supplemented with 50 μg/mL hygromycin and confirmed through PCR, then further confirmed by qRT-PCR and Southern blot analysis [36,37].

For constructing the complementary strain of the Δ*Apwd40a* mutant, the full-length *ApWD40a* coding region, containing 1.2–1.5 kb upstream as a promoter and 528 bp downstream as a terminator, was ligated into the vector pCAMBIA1300_*SUR* (*Xba* I and *Hin*d III) by seamless cloning. The plasmid was subsequently transferred into the *Agrobacterium tumefaciens* AGL-1 strain. The complementation vector was introduced into the protoplasts of the Δ*Apwd40a* mutant via *Agrobacterium tumefaciens*-mediated transformation (ATMT) to complement the deleted *ApWD40a* gene in the Δ*Apwd40a* mutant. Putative positive transformants, capable of growing on sulfonylurea (10 µg/mL)-supplemented DCM, were validated by PCR and further confirmed by qRT-PCR.

Using the same approach, we constructed knockout mutants for the differentially expressed gene (DEG) *ApSulP2*, which was screened from the DEGs by RNA-seq. A comprehensive list of all primers employed in this research is provided in Supplementary Table S1.

### 2.3. Genetic Manipulation

For DNA and RNA isolation, 3–5 agar plugs were inoculated into PDB and cultivated at 25 °C and 180 rpm oscillation for 4 d. For the Southern blotting, genomic DNA was extracted from the JY15 strain and the null mutant, digested with *Eco*R V, and subjected to 0.7% agarose gel electrophoresis. The detached fragments were transferred onto Hybond N+-labeled membranes (Amersham Biosciences, Buckinghamshire, UK) and visualized using the standard protocol for the DIG High Prime DNA Labeling and Detection Kit (Roche Diagnostics, Mannheim, Germany).

### 2.4. Real-Time Quantitative PCR

Total RNA was extracted using an OminiPlant RNA Kit (CoWin Biotech, Beijing, China). Reverse transcription PCR and qRT-PCR were performed using the standard protocol for the 2×NovoScript^®^ Plus 1st Strand cDNA Synthesis SuperMix and 2×NovoStart^®^ SYBR qPCR SuperMix Plus, respectively (Novoprotein, Suzhou, China). The qPCR reactions were conducted on a LightCycler 96 real-time PCR system (Roche, Mannheim, Germany) using gene-specific primers and qPCR SuperMix Plus, with three replicates for each experiment. The qPCR data were normalized using *RPB2* as an internal control, and the relative expression was calculated using the 2^−ΔΔCt^ method [38,39].

### 2.5. Assays for Vegetative Growth and Utilization of Different Carbon Sources

Mycelia plugs (6 mm in diameter) from 7-day-old PDA were inoculated onto 90 mm Petri dishes containing PDA, V8, CM, MM, or OA media. The radial growth of vegetative mycelia was measured after 8 d of cultivation, and the sporulation was assessed by culturing different *A. panax* strains on V8 juice agar and incubating them at 25 °C for 15 d. Conidia were collected by gently scraping the mycelial surface with a sterile spreader, resuspended in ddH_2_O, and counted using a hemocytometer. Carbon source utilization assays for each strain were performed by measuring the growth on MM containing different sole carbon sources, i.e., 2% glucose, 2% sucrose, 2% glycerol, 2% starch, and 2% xylose, at 25 °C for 8 d [30].

### 2.6. Growth Under Different Stress Conditions

Stress response assays were performed by culturing *A. panax* strains on MM containing 0.2 mg/mL Congo red, 0.01% SDS, 1 M sorbitol, 0.7 M NaCl, 0.6 M KCl, 10 mM H_2_O_2_, or 3 mM paraquat after 8 d of incubation at 25 °C [34]. Colony diameter were recorded, and the mycelial growth inhibition rate was calculated as previous methods [37].

### 2.7. Penetration Ability and Surface Hydrophobicity Assays

The penetration ability and surface hydrophobicity of the *A. panax* strains were determined according to a previously described method [40]. Mycelial plugs of the tested strains were positioned onto cellophane membranes on PDA and cultured at 25 °C for 96 h. After the cellophane membranes with fungal colonies were removed, the remaining PDA was incubated at 25 °C for 72 h to observe the growth of mycelia that had penetrated through. The colony surface hydrophobicity was assessed by culturing the *A. panax* strains on PDA for 10 d. Twenty microliters each of ddH_2_O and 5 mM EDTA + 0.02% SDS solution were dropped onto the culture surface of each strain. Then, the hydrophobicity was recorded after 12 h of incubation. Each experiment was performed as three independent replicates.

### 2.8. Determination of Melanin Content

The wild-type JY15 and the Δ*Apwd40a* and Δ*Apwd40a-C* mutants were cultured on PDA for 7 days; then, five mycelial plugs were inoculated into 100 mL of PDB, and the cultures were kept in darkness at 25 °C and 150 rpm oscillation for 5 d. One gram of (wet weight) mycelium was weighed separately for each sample, treated with 10 mL of 0.5 M NaOH, and boiled in water (100 °C) for 2 h. After boiling, the sample was collected by centrifugation, adjusted to a pH of 2.0 by the addition of 5 M HCl, and followed by centrifugation at 6000× *g* for 15 min at ambient temperature. The resulting pellet was gathered, rinsed three times using distilled water, and then resuspended in a solution of 0.5 M NaOH. The absorbance of the solution was determined at 459 nm, with 0.5 M NaOH serving as a reference control [41]. Each experiment was performed with three independent replicates.

### 2.9. Determination of Mycotoxin Tyrosol

Tyrosol was extracted from fungal cultures as described [9]. The wild-type JY15 and the Δ*Apwd40a* mutant strain were each cultured on PDA for 7 d. Six mycelial plugs (8 mm in diameter) were then removed from the edge of the colony, inoculated into 100 mL of PDB (500 mL each), and incubated for 2 weeks with shaking at 25 °C and 150 rpm. The mycelia were separated by passing them through a triple layer of lens paper. The culture filtrates were subjected to extraction with ethyl acetate three times (1:1, *v*/*v*), after which the ethyl acetate fractions were combined, and the solvent from the combined organic phase was removed by evaporation at 45 °C under reduced pressure. After concentrating, an appropriate amount of chromatographic-grade methanol was added to dissolve the solution. Tyrosol was separated on a BDS HYPERSIL C18 (250 mm × 4.6 mm) attached to an Agilent 1260 LC HPLC system using methanol/H_2_O as a mobile phase at a flow rate of 0.8 mL/min and a column temperature of 35 °C. Tyrosol was detected by a UV detector with absorbance measured at 275 nm.

### 2.10. Pathogenicity Analysis

Pathogenicity assays of the *A. panax* strains were conducted following previously reported protocols [5]. The wild-type JY15, the Δ*Apwd40a* mutant, and the Δ*Apwd40a-C* mutant were grown on PDA at 25 °C for 7 d. Agar plugs of 6 mm diameter (mycelia side down) were inoculated onto detached leaves and roots of healthy ginseng plants separately. The leaves and roots were gently pricked with a fine sterilized needle before inoculation to simulate inoculated leaf and root injuries. Pathogenicity tests were conducted on five leaves and roots of each strain separately. Finally, the inoculated leaves and roots were individually positioned in a sterile, moist box with two layers of sterilized filter paper and maintained at 25 °C under a photoperiod of 12 h of light followed by 12 h of darkness for 7 d. Disease symptoms were observed and recorded after 7 d inoculation. PDA plugs served as the negative control. Each pathogenicity experiment was performed for at least five replicates.

### 2.11. Transcriptome Sequencing

The wild-type JY15 and Δ*Apwd40a* strains were cultured on PDB under dark conditions at 25 °C in a shaking culture for 7 d, and the mycelia were collected by filtration and quickly frozen in liquid nitrogen and stored. RNA sequencing was conducted utilizing three biological replicates per sample, with the samples dispatched to NovoGene Co., Ltd., Beijing, China for analysis. The RNA integrity was evaluated with the RNA Nano 6000 Assay Kit on the Bioanalyzer 2100 System (Agilent Technologies, Foster City, CA, USA). Libraries for sequencing were constructed with the NEBNext^®^ Ultra™ RNA Library Preparation Kit for Illumina^®^ (New England Biolabs, Inc., Ipswich, MA, USA) and subsequently sequenced on an Illumina Novaseq instrument, producing 150 bp paired-end sequences. Clean data (clean reads), comprising high-quality reads, were derived from the raw data by eliminating sequences with adapter contamination, stretches of poly-N, and those identified as being of low quality. Subsequent analyses relied exclusively on the high-quality reads. These clean reads were mapped onto the *A. panax* BNCC115425 reference genome utilizing the HISAT2 software version 2.0.5, and gene-specific read counts were determined by featureCounts version 1.5.0-p3 [42,43].

### 2.12. DEG Analysis

The FPKM (Fragments Per Kilobase of transcript per Million mapped reads) values for each gene were determined by considering both the gene’s sequence length and the quantity of reads aligned to it. DESeqR software version 1.20.0 was utilized to analyze the differential expression in digital gene expression data between the wild-type JY15 and the Δ*Apwd40a* strains [44].

### 2.13. GO and KEGG Enrichment Analysis of DEGs

The functional categorization and pathway analysis of the DEGs were performed using the ClusterProfiler R package version 3.8.1, with a gene length bias correction applied to ensure accuracy. Significant GO terms among the DEGs were identified with a corrected *p*-value threshold set at less than 0.05. The KEGG is a comprehensive resource that facilitates the understanding of biological systems’ complex functions and roles at the cellular, organismal, and ecosystem levels. It integrates molecular-level data, particularly from extensive molecular datasets derived from genome sequencing and high-throughput experimental technologies, into a cohesive framework for biological insight. Additionally, gene clusters related to secondary metabolites were predicted using antiSMASH 7.0 [45,46].

### 2.14. Determination of Biomass

Biomass, represented by DNA content, was determined using the TCA method. One milliliter of fungal mycelia suspension of the tested strains was diluted 5-fold by adding 4 mL of ddH_2_O water into 15 mL centrifuge tubes and centrifuged for 12 h at 4 °C and 1000× *g*. The supernatant was discarded, and 1 mL of 10% TCA was added to the centrifuge tubes and kept on ice for 3 min and repeated once. The tubes were placed in a boiling water bath for 30 min, the supernatant was removed by centrifugation, and the DNA concentration was measured using a NanoDrop 2000 (Thermo Fisher Scientific, Waltham, MA, USA). Each assay was performed for at least three replicates.

### 2.15. Statistical Analysis

Statistical analysis was performed using IBM SPSS Statistics 26. The significance of the between-sample differences for all experimental data was determined via Tukey’s honestly significant difference test (*p* < 0.05).

## 3. Results

### 3.1. Identification of ApWD40a and Targeted Gene Disruption of ApWD40a in A. panax

**Figure 1:**
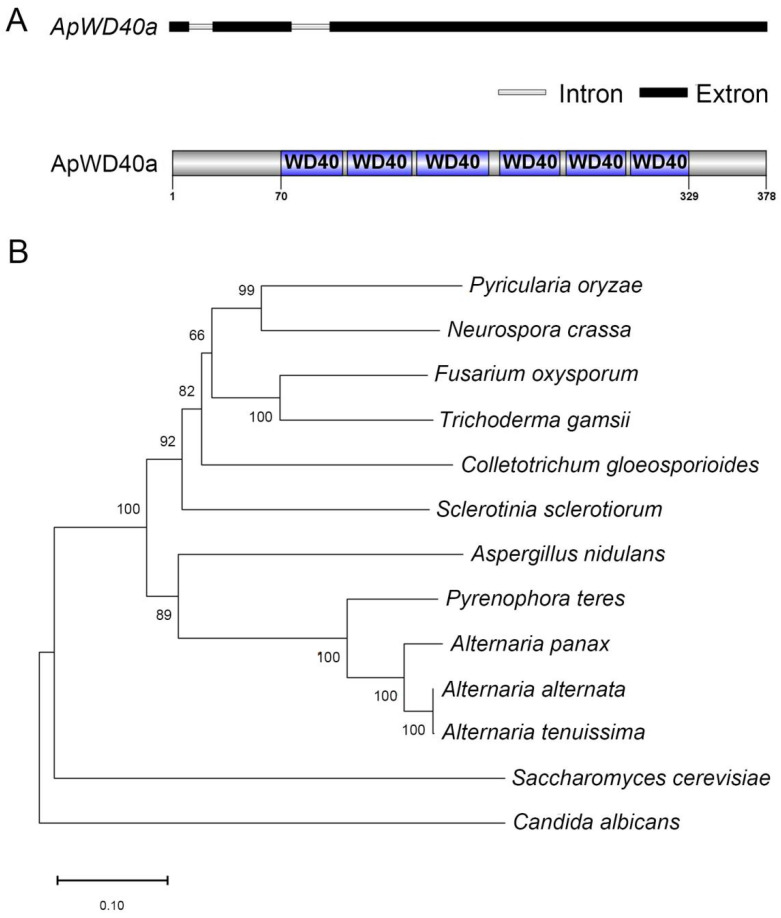
Structure analysis of ApWD40a in *A. panax*. (**A**) Primary structure of ApWD40a predicted by SMART. The WD40 repeat domains are indicated in blue. (**B**) Phylogenetic tree analysis of ApWD40a proteins. The following WD40a proteins were utilized for the construction of the phylogenetic tree: *Magnaporthe oryzae* XP_003709713.1; *Neurospora crassa* XP_963156.3; *Fusarium oxysporum* EGU75440.1; *Trichoderma gamsii* XP_018663274.1; *Colletotrichum gloeosporioides* EQB52344.1; *Sclerotinia sclerotiorum* XP_001597417.1; *Aspergillus nidulans* XP_658660.1; *Pyrenophora teres* EFQ89095.1; *Alternaria panax* JY15; *Alternaria alternata* KAH6846796.1; *Alternaria tenuissima* RYN38094.1; *Saccharomyces cerevisiae* NP_012218.1; *Candida albicans* XP_717966.1.

Based on the whole-genome database information for *A. panax* BNCC115425 (accession number: AANER000000000) combined with the PHI database (http://www.phi-base.org/index.jsp (accessed on 20 August 2021)), we identified a pathogenicity-related gene belonging to the WD40 family, which we named *ApWD40a*. The *ApWD40a* gene was found to be 1270 bp in length, with two intron regions, and encoded 378 amino acids (Figure 1A). The primary structure analysis indicated that the protein contained six repeated WD40 structures (~40 amino acids each). The multiple sequence alignment results across seven fungal species showed that several sites within the WD40 domain were highly conserved (Figure S1). Phylogenetic analysis indicated that ApWD40a was most closely affiliated with *A. alternata* and *A. tenuissima*, while it was more distantly related to *Candida albicans* and *S. cerevisiae* (Figure 1B). The results show that the WD40a protein was widely distributed and highly conserved in filamentous fungi.

To further investigate the role of the *ApWD40a* gene in *A. panax*, a seamless cloning method was employed to construct a knockout vector for the targeted gene (Figure S2A). The constructed vector was transformed into *A. panax* by the ATMT method. One knockout mutant of the target gene Δ*Apwd40a* was obtained and verified by Southern blot analysis (Figure S2B). To generate complemented transformants, the complementation vector pCAMBIA1300_*SUR_WD40a* was reintroduced into the Δ*Apwd40*a mutant via an ATMT. The complemented transformant Δ*Apwd40a-C* was confirmed by PCR and qRT-PCR. Phenotypic consistency was observed between the wild-type JY15 and the complemented transformant Δ*Apwd40a-C* compared with the null mutant Δ*Apwd40a* for subsequent experiments.

### 3.2. Deletion of ApWD40a Resulted in Defective Vegetative Growth of A. panax

**Figure 2:**
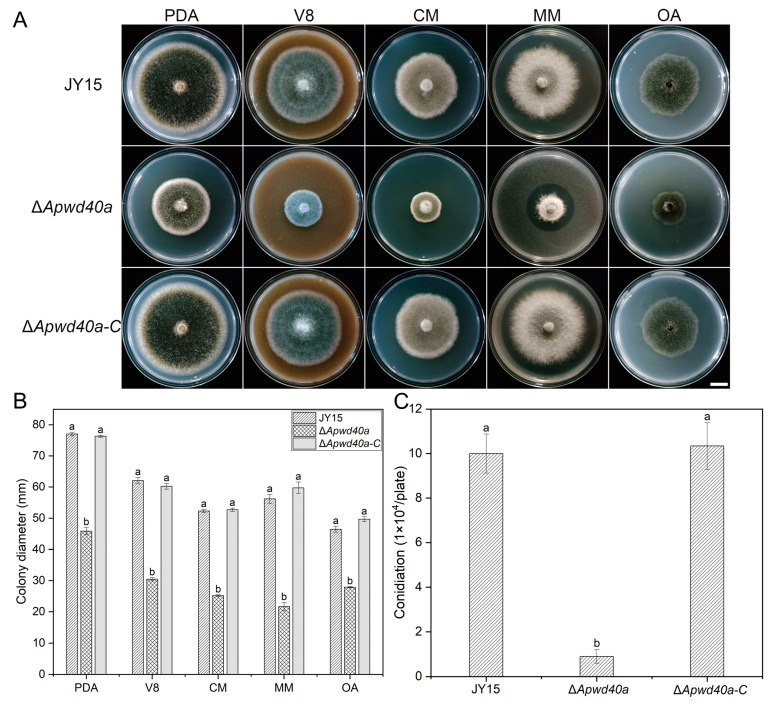
Effects of the *ApWD40a* gene on the mycelial growth and conidial production of *A. panax*. (**A**) Colony characteristics of the wild-type JY15, Δ*Apwd40a*, and Δ*Apwd40a-C* on PDA, V8, CM, MM, or OA media after 8 d of cultivation at 25 °C. (**B**) Colony diameter of the wild-type JY15, Δ*Apwd40a*, and Δ*Apwd40a-C* on different media after 8 d of cultivation at 25 °C. (**C**) Conidial production of the wild-type JY15, Δ*Apwd40a*, and Δ*Apwd40a-C* on V8 after 15 d of cultivation at 25 °C. Each experiment was performed for three independent replicates. The error bars represent the standard deviations. Distinct lowercase letters denote statistically significant differences at the *p* < 0.05 threshold, as determined by Tukey’s honestly significant difference test. Bar: 1 cm.

To investigate whether *ApWD40a* is involved in the vegetative growth of *A. panax*, we measured the growth rates of the wild-type JY15, the Δ*Apwd40*a mutant, and the complemented strain Δ*Apwd40a-C* on five media. The results indicate that the Δ*Apwd40*a mutant exhibited 41%, 51%, 51.2%, 61.5%, and 39.9% reductions in the mycelial growth compared with the wild-type JY15 on PDA, V8, CM, MM, and OA media, respectively (Figure 2A,B). Moreover, the conidial production was significantly decreased by 90% after cultivation for 15 d on the V8 medium (Figure 2C). The phenotypic defects observed in the Δ*Apwd40a* mutant were fully restored in the complemented strain Δ*Apwd40a-C*, demonstrating that the defects were caused by the deletion of *ApWD40a*. These results suggest that *ApWD40a* played a significant role in the vegetative growth and conidiation of *A. panax*.

### 3.3. ApWD40a Was Involved in Carbon Source Utilization of A. panax

**Figure 3:**
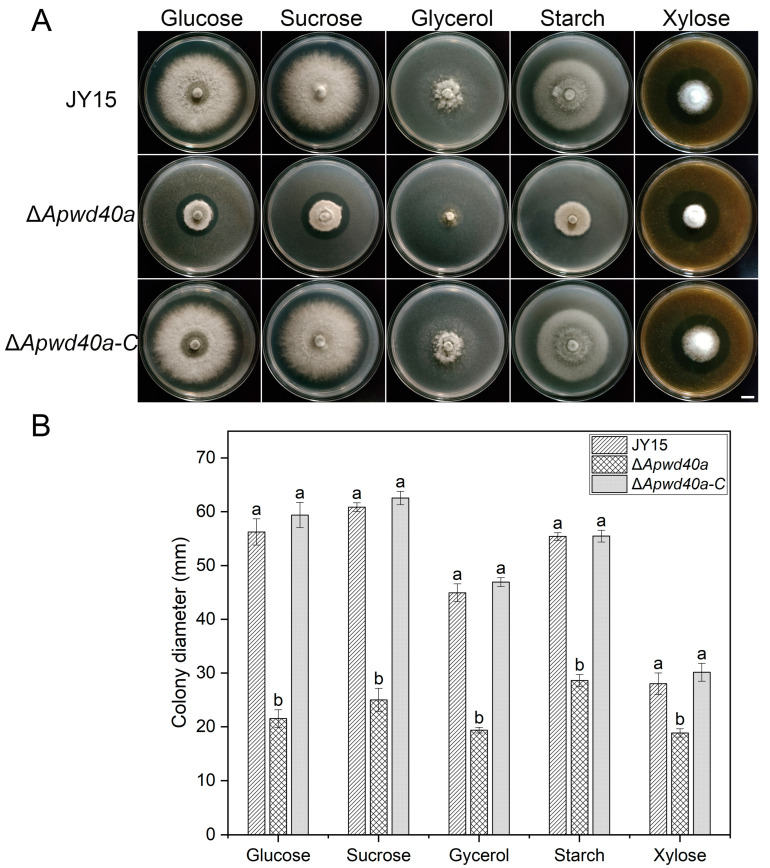
Carbon source utilization of the wild-type JY15, Δ*Apwd40a*, and Δ*Apwd40a-C* strains. (**A**) Colony characteristics of different strains on MM supplemented with 2% glucose, 2% sucrose, 2% glycerol, 2% starch, and 2% xylose after 8 d of cultivation at 25 °C. (**B**) Colony diameter of the wild-type JY15 and its derivative strains on MM with assorted carbon substrates. Each experiment was performed for three independent replicates. The error bars represent the standard deviations. Distinct lowercase letters denote statistically significant differences at the *p* < 0.05 threshold, as determined by Tukey’s honestly significant difference test. Bar: 1 cm.

A variety of carbon sources serve as nutritional elements for fungi. However, different carbon sources provide different levels of nutrition, which, in turn, can lead to differences in the growth and development of pathogenic fungi. To further investigate whether *ApWD40a* is involved in carbon source utilization, this study examined the utilization efficiency of the *ApWD40a* mutant for 2% glucose, 2% sucrose, 2% glycerol, 2% starch, and 2% xylose by using MM containing each of these carbon sources. By measuring the colony diameter, it was found that the growth rate of Δ*Apwd40*a on these carbon sources was still lower than that of the wild-type JY15 (Figure 3A,B). The most significant decreases in the growth rate were discovered on glucose and sucrose, followed by glycerol and starch, as the sole carbon sources. We thus concluded that *ApWD40a* affected the carbon source utilization ability of *A. panax*.

### 3.4. ApWD40a Played Various Roles in Stress Responses of A. panax

**Figure 4:**
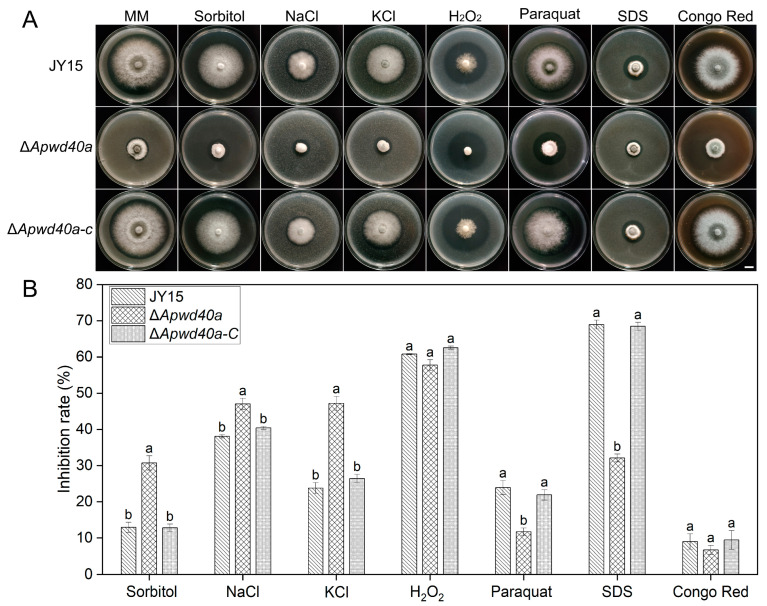
Roles of the *ApWD40a* gene in different stress responses of *A. panax* strains. (**A**) Colony characteristics of the wild-type JY15, Δ*Apwd40a*, and Δ*Apwd40a-C* on MM supplemented with sorbitol (1 M), NaCl (0.7 M), KCl (0.6 M), H_2_O_2_ (10 mM), paraquat (3 mM), Congo red (0.2 mg/mL), or SDS (0.01%) after 8 d of cultivation at 25 °C. (**B**) Growth inhibition rates of the wild-type JY15, Δ*Apwd40a*, and Δ*Apwd40a-C* on MM with various stress-inducing agents. Each experiment was performed for three independent replicates. The error bars represent the standard deviations. Distinct lowercase letters denote statistically significant differences at the *p* < 0.05 threshold, as determined by Tukey’s honestly significant difference test. Bar: 1 cm.

Plant pathogenic fungi face various environmental stresses during their growth, such as osmotic stress, metal ion stress, and oxidative stress [47]. To explore the role of *ApWD40a* in response to different external stresses, we measured the growth rates of the wild-type JY15, the Δ*Apwd40*a mutant, and the complemented strain Δ*Apwd40a-C* on MM supplemented with the osmotic stress factor sorbitol, the metal-ion-containing compounds NaCl and KCl, the oxidative stress factors H_2_O_2_ and paraquat, and the cell-wall-perturbing agents Congo red and SDS. The results indicate that compared with the wild-type JY15, the mutant Δ*Apwd40a* exhibited an increased sensitivity to sorbitol, KCl, and NaCl, with an increased inhibition rate of mycelia growth (Figure 4A,B). In contrast, Δ*Apwd40*a showed a decreased sensitivity to paraquat. The inhibition rates of mycelia growth on MM supplemented with H_2_O_2_ or SDS were decreased, but not significantly (Figure 4A,B).

### 3.5. ApWD40a Was Important for the Pathogenicity of A. panax

**Figure 5:**
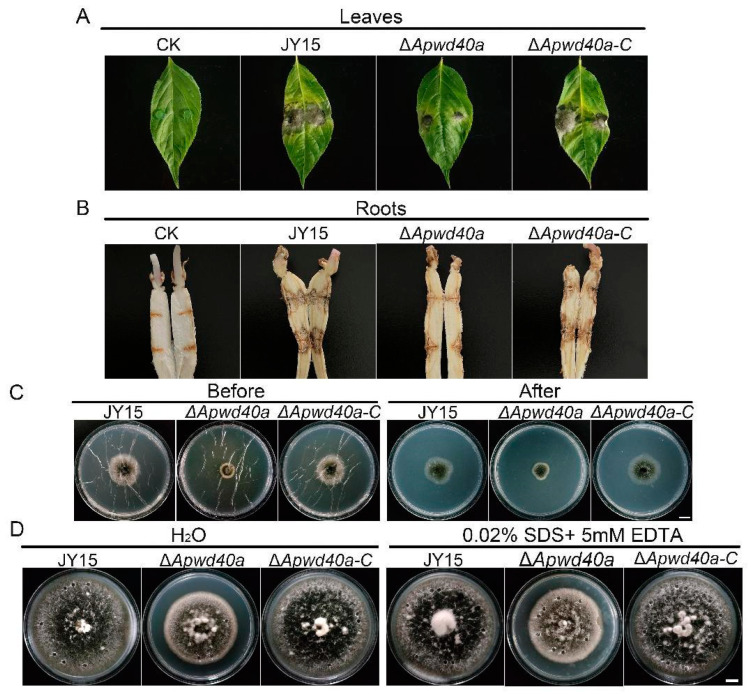
Pathogenicity assays of *A. panax* strains. (**A**) Virulence assays on detached ginseng leaves. Agar plugs of the wild-type JY15, Δ*Apwd40a*, and Δ*Apwd40a-C* strains with grown mycelia were inoculated on ginseng leaves and cultured in a moist chamber for 7 d. PDA agar plugs were used as the control. (**B**) Virulence assays on ginseng roots. Agar plugs of the wild-type Y15, Δ*Apwd40a*, and Δ*Apwd40a-C* strains with grown mycelia were inoculated on ginseng roots and cultured in a moist chamber for 7 d at 25 °C. The ginseng roots were cut along their length and recorded. PDA plugs were used as the control. Each experiment was performed for three independent replicates. (**C**) Penetration ability of *A. panax* strains against a cellophane membrane. Fungal discs of the strains were inoculated on MM plates covered with cellophane membranes and cultivated at 25 °C for 4 d. Following the removal of the membranes and the mycelial inoculants, the plates were incubated at 25 °C for an additional 3 d to assess the colonization of the mycelium that had penetrated the media. (**D**) Hydrophobicity test of *A. panax* strains. The hydrophobicity of the wild-type JY15, Δ*Apwd40a*, and Δ*Apwd40a-C* strains was assessed by placing 20 μL of a solution containing 0.02% SDS and 0.5 mM EDTA on the colony surface. Photographs were recorded after 12 h of incubation. Each experiment was performed for three independent replicates. Bar: 1 cm.

To evaluate the possible role of *ApWD40a* in ginseng pathogenicity, we assessed the pathogenicity of the Δ*Apwd40a* mutant against ginseng leaves and roots. The virulence of the fungal strains was tested by inoculating wounded ginseng leaves and roots with wild-type JY15, Δ*Apwd40a*, and Δ*Apwd40a-C*, followed by the observation of disease symptoms after 7 d. The results indicate that compared with the wild-type JY15 and Δ*Apwd40a-C*, the pathogenicity of Δ*Apwd40a* against ginseng leaves and roots was significantly reduced, with only water-soaked areas on the leaves and smaller lesion areas on the roots observed, along with lighter-colored lesions (Figure 5A,B). These findings suggest that the deletion of *ApWD40a* led to reduced virulence, and *ApWD40a* was required for the full pathogenicity of *A. panax* against ginseng.

### 3.6. ApWD40a Did Not Affect Penetration Ability or Hydrophobicity of A. panax

We next conducted penetration ability assays and colony surface hydrophobicity tests to investigate the changes in the penetration ability and hydrophobicity of *ApWD40a* (Figure 5C,D). As shown in Figure 5C, in contrast to the wild-type JY15 and complemented strain Δ*Apwd40a-C*, the Δ*Apwd40a* mutant was still able to penetrate the cellophane membrane and grow on PDA. Similarly, the hydrophobicity test showed that water and detergent droplets remained on the colony surface without spreading into the mycelia of the Δ*Apwd40a* mutant after a 12 h of incubation. No significant differences were observed between the wild-type JY15 and the Δ*Apwd40a* mutant. These findings imply that the role of *ApWD40a* in pathogenicity might not directly involve changes in the penetration ability or hydrophobicity of *A. panax.*

### 3.7. Melanin Synthesis Was Impaired in A. panax

**Figure 6:**
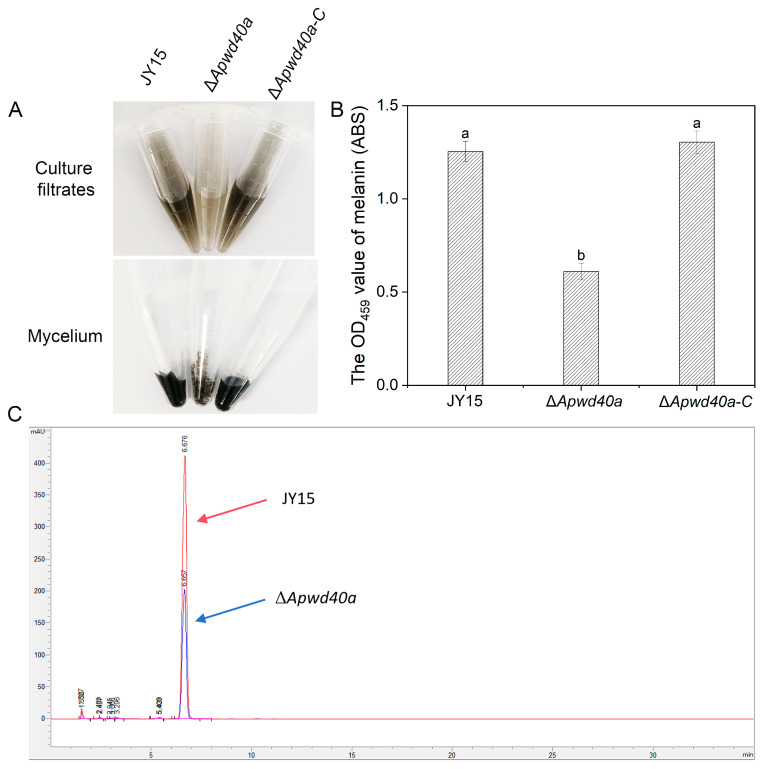
Melanin accumulation and toxin determination of *A. panax* strains. (**A**) Melanin accumulation in *A. panax* strains grown in PDB. (**B**) Melanin content in the mycelia of the wild-type JY15, Δ*Apwd40a*, and Δ*Apwd40a-C* strains. (**C**) HPLC analysis of the *A. panax* toxin tyrosol. Arrows indicate the tyrosol peaks for the wild-type JY15 (red) and the Δ*Apwd40a* mutant (blue). Each experiment was performed for three independent replicates. The error bars represent the standard deviations. Distinct lowercase letters denote statistically significant differences at the *p* < 0.05 threshold, as determined by Tukey’s honestly significant difference test.

Melanin plays important roles in various aspects of fungal growth, development, and pathogenicity [48]. To explore whether the deletion of *ApWD40a* affects melanin synthesis, we measured the melanin contents in the mycelium of the wild-type JY15, the Δ*Apwd40a* mutant, and the complemented strain Δ*Apwd40a-C*. After culturing in PDB for 5 d, the mycelia were filtered to obtain the fermentation broth for subsequent determination. As shown in Figure 6A, the fermentation broth and mycelial pigmentation of the Δ*Apwd40a* mutant were lighter than those of the wild-type JY15, although some pigment was still produced. The melanin content of Δ*Apwd40a* was significantly reduced by 51.2% compared with the wild-type JY15, suggesting that the deletion of the *ApWD40a* significantly affected the synthesis of melanin in *A. panax* (Figure 6B).

### 3.8. ApWD40a Was Crucial for Toxin Synthesis of A. panax

Toxins are known to be among the significant virulence factors in *Alternaria* spp., with nearly 20 host-specific toxins reported to date. In 2024, the targeted isolation of mycotoxins in *A. panax* identified three small molecule compound toxins, namely, tyrosol, 3-hydroxy-3-(4-methoxyphenyl) propanoic acid, and 3-benzylpiperazine-2,5-dione. Toxicity assays on ginseng leaves revealed that tyrosol exhibited the strongest toxicity [9]. To further investigate whether the deletion of *ApWD40a* affects the synthesis of tyrosol, we cultured the wild-type JY15 and Δ*Apwd40a* in PDB for 15 d to produce toxins. The crude toxins were extracted with filtrate and an equal volume of ethyl acetate three times, concentrated to near dryness using a rotary evaporator, and dissolved in chromatography-grade methanol. The HPLC results showed that the content of tyrosol in the Δ*Apwd40a* mutant was significantly lower than in the wild-type JY15 (Figure 6C), demonstrating that the involvement of *ApWD40a* in the pathogenicity of *A. panax* may have been regulated by the synthesis of secondary metabolites, such as melanin and toxins.

### 3.9. Comparative Transcriptome Analysis Revealed the Comprehensive Regulatory Functions That Defined the Global Regulatory Role of ApWD40a in A. panax

**Figure 7:**
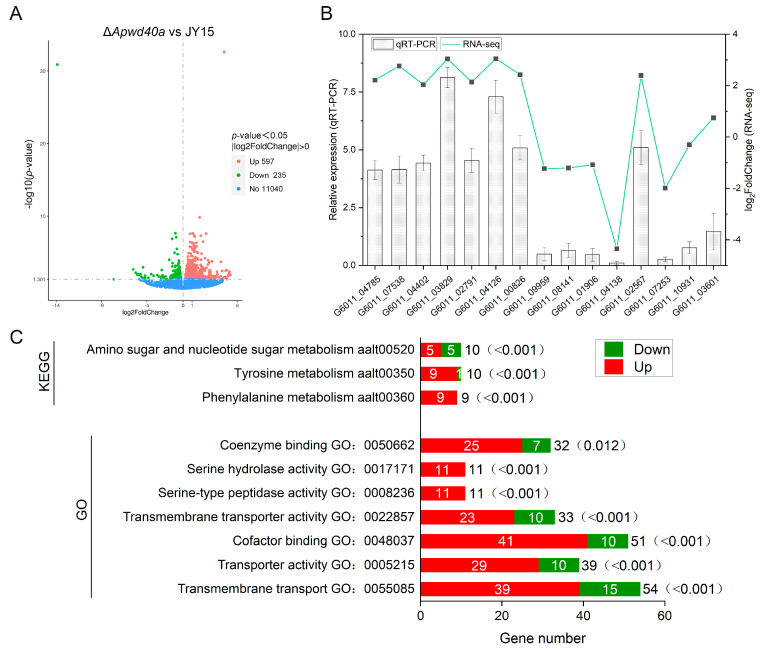
Differential transcriptome analysis between the wild-type JY15 and the Δ*Apwd40a* mutant. (**A**) A graphical representation of gene expression disparities, highlighting the contrast between the wild-type JY15 and the Δ*Apwd40a* mutant, is depicted in a volcano plot. In this plot, the horizontal axis corresponds to the log_2_FoldChange of the gene expression, while the vertical axis corresponds to the −log_10_(*p*-value), signifying statistical significance. Genes that exhibited significant upregulation [log_2_FoldChange > 0 and *p*-value < 0.05] or downregulation [log_2_FoldChange < 0 and *p*-value < 0.05] in the Δ*Apwd40a* mutant are marked with red and green dots, respectively. Genes that remained unchanged are depicted as light blue dots. (**B**) qRT-PCR was conducted to affirm the expression patterns of 15 DEGs randomly selected from the Δ*Apwd40a* mutant strain. (**C**) Results of the GO and KEGG enrichment analyses of DEGs between the wild-type JY15 and the Δ*Apwd40a* mutant. The number of DEGs is shown next to the columns, with the *p*-values indicated in parentheses. Each qRT-PCR experiment was performed for three independent replicates. The error bars represent the standard deviations.

To further explore the regulatory mechanisms associated with *ApWD40a*, a comparative transcriptome analysis was performed between the wild-type JY15 and the mutant Δ*Apwd40a* in *A. panax*. The genome of the wild-type strain BNCC115425 (accession number: JAANER000000000) was used as the reference. Over 95% of the reads from all six samples mapped to the reference genome, and the proportion of reads that matched the exon regions of the reference genome was above 80% (Figure S3A), which is relatively high. The correlation coefficient between the wild-type JY15 and the Δ*Apwd40a* mutant was higher than 0.95 (Figure S3B). The RNA-seq results identified a total of 832 DEGs [|log_2_(FoldChange)| > 0 and *p*-value < 0.05] (Figure 7A), which represented approximately 28.2% of the entire *A. panax* genome. These DEGs comprised 597 upregulated genes and 235 downregulated genes. To further validate the reliability of the data, 15 candidate genes were selected for qRT-PCR verification based on the DEG identification results from the transcriptome sequencing (Figure 7B, Table S1). The results reveal that the transcript levels of genes verified by qRT-PCR were consistent with those from the RNA-seq.

To elucidate the specific gene roles regulated by the *ApWD40a* in *A. panax*, we initially conducted GO enrichment analysis to assess the functions of DEGs in the Δ*Apwd40a* mutant. The enriched DEGs were involved in transmembrane transport, cofactor binding, transporter activity, transmembrane transporter activity, coenzyme binding, and carbohydrate metabolic processes. The KEGG enrichment analysis suggested that *ApWD40a* potentially exerted its influence on the gene expression within the metabolic pathways of phenylalanine, tyrosine, and amino sugars and nucleotide sugars (Figure 7C). Furthermore, *ApWD40a* affected the glutathione metabolism, carbon metabolism, and various other metabolic pathways. Based on the results of the enrichment analysis, we created scatter plots of the 30 most significant GO terms and 20 most significant KEGG terms to predict which genes may be regulated by *ApWD40a* (Figures S4 and S5).

**Figure 8:**
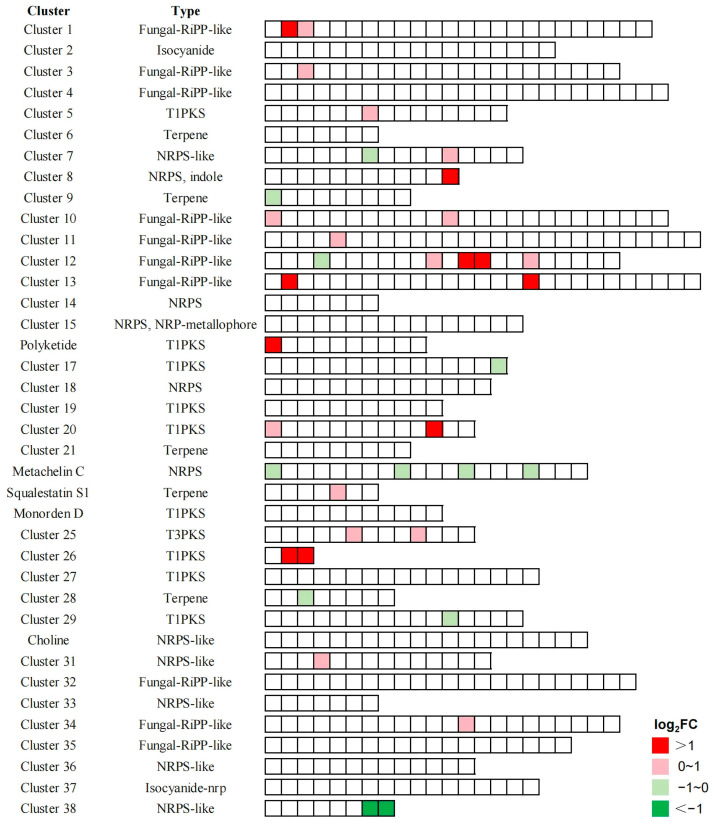
The differential expression patterns of gene clusters related to secondary metabolite biosynthesis in the Δ*Apwd40a* mutant. Each box represents an individual gene. Genes that exhibited a significant increase in expression, characterized by log_2_FoldChange > 1 and *p-*value < 0.05, are denoted by red dots; conversely, those that showed a significant decrease in expression, with a log_2_FoldChange < −1 and *p-*value < 0.05, are indicated by green dots. Genes with moderate upregulation [log_2_FoldChange > 0 and *p-*value < 0.05] and downregulation [log_2_FoldChange < 0 and *p-*value < 0.05] are represented by pink and light green dots, respectively.

To investigate whether *ApWD40a* affects the expression of genes related to the synthesis of secondary metabolites in *A. panax*, we utilized antiSMASH 7.0 [46] to predict the transcriptional regulation of biosynthetic gene clusters within this fungus. The results show that *ApWD40a* regulated a total of 38 gene clusters related to secondary metabolite biosynthesis. By mapping these genes onto the transcriptome data, we observed significant changes in the expression of genes within 22 secondary metabolite gene clusters in the Δ*Apwd40a* mutant. In Clusters 1, 10, 11, 13, 20, 25, and 26, two or more genes showed significant upregulation, with no downregulated genes detected, suggesting that *ApWD40a* may have negatively regulated the transcription of these seven gene clusters. In Clusters 9, 17, metachelin C, 28, 29, and 38, the secondary metabolite-related genes all exhibited significant downregulation, with the highest number of downregulated genes in the metachelin C cluster and no upregulated genes detected. These results suggest that *ApWD40a* may have positively regulated the transcription of these six gene clusters. Clusters 7 and 12 contained both upregulated and downregulated DEGs, with more upregulated genes in Cluster 12, indicating relatively complex transcriptional regulation by the Δ*Apwd40a* mutant within these two gene clusters (Figure 8).

### 3.10. ApSulP2 Participated in Sulfate Transport but Did Not Influence Pathogenicity of A. panax

**Figure 9:**
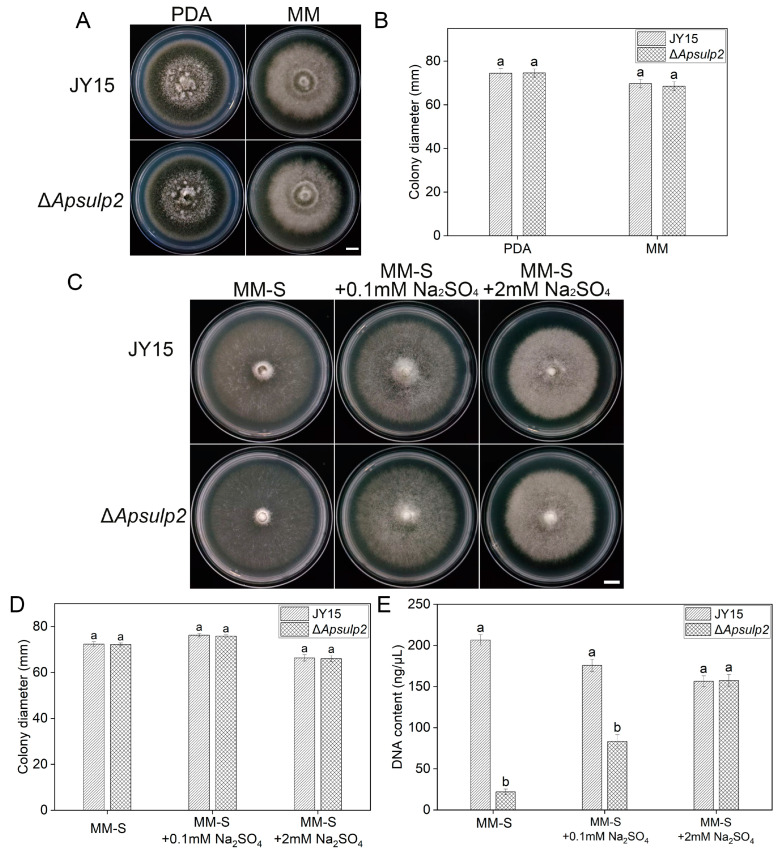
Different effects of *ApSulP2* on mycelia growth and sulfate utilization of *A. panax*. (**A**) Colony characteristics of the wild-type JY15 and Δ*Apsulp2* on PDA and regular MM after an 8 d cultivation. (**B**) Colony diameter of the wild-type JY15 and Δ*Apsulp2* on PDA and regular MM. (**C**) Colony characteristics of the wild-type JY15 and Δ*Apsulp2* on sulfate-free MM (MM-S) plates and MM-S with low (0.1 mM Na_2_SO_4_) and high (2 mM Na_2_SO_4_) doses of sulfate. (**D**) Colony diameter of the wild-type JY15 and Δ*Apsulp2* on MM-S plates and MM-S with low (0.1 mM Na_2_SO_4_) and high (2 mM Na_2_SO_4_) doses of sulfate. (**E**) Biomass of the wild-type JY15 and Δ*Apsulp2* on liquid MM-S and MM-S with low (0.1 mM Na_2_SO_4_) and high (2 mM Na_2_SO_4_) doses of sulfate. The biomasses of different strains were determined by the TCA method and in terms of the DNA content. Each experiment (**B**,**D**,**E**) was performed for three independent replicates. The error bars represent the standard deviations. Distinct lowercase letters denote statistically significant differences at the *p* < 0.05 threshold, as determined by Tukey’s honestly significant difference test. Bar: 1 cm.

Transcriptome analysis based on RNA-seq identified the G6011_04785 gene, which was significantly upregulated in the Δ*Apwd40a* mutant (Table S5) and further confirmed by qRT-PCR. The G6011_04785 gene was predicted by BLASTp to encode sulfate permease 2 and was named *ApSulP2* for further study. *ApSulP2* consisted of 678 amino acids with a total molecular weight of 74.25 kDa and contained two domains, Sulfate_transp and STAS (Figure S6A), which showed 38.2% identity with the sulfate permease 2 protein of *A. nidulans* (Figure S6B). To elucidate the role of *ApSulP2* and the possible relationship between *ApWD40a* and *ApSulP2* in *A. panax*, a targeted deletion mutant of *ApSulP2* was constructed by homologous recombination (Figure S7A). The Δ*Apsulp*2 mutant exhibited a growth rate comparable with that of the wild-type JY15 on both PDA and conventional MM (Figure 9A,B). Sulfate permease has been reported to be involved in sulfate uptake by *Penicillium chrysogenum* and *A. nidulans* [49,50]. Thus, to elucidate the role of *ApSulP2* in affecting the absorption and utilization of sulfate, the wild-type JY15 and the Δ*Apsulp2* mutant were inoculated on sulfate-free MM (MM-S) and cultivated at 25 °C for 8 d. The growth rates of Δ*Apsulp2* and the wild-type JY15 were similar, while the Δ*Apsulp2* mutant formed a very sparse mycelial layer on MM-S, which was restored with the addition of inorganic sulfate (Figure 9C,D). To further validate these findings, the wild-type JY15 and the Δ*Apsulp2* mutant were cultured in liquid MM-S and in MM-S supplemented with 0.1 mM Na_2_SO_4_ or 2 mM Na_2_SO_4_ for 15 d, and their biomasses were estimated using the DNA content. The results indicate that in the absence of sulfate, the biomass of the Δ*Apsulp2* mutant was approximately 21.9 ng/μL, which is approximately one-tenth that of the wild-type JY15. Upon the addition of 0.1 mM (low inorganic sulfate concentration) Na_2_SO_4_ and 2 mM (high inorganic sulfate concentration) Na_2_SO_4_ to the MM-S medium, the biomasses of the Δ*Apsulp2* mutant increased to 83.3 ng/μL and 157.2 ng/μL, respectively (Figure 9E). These biomass measurements further confirmed that the absence of *ApSulP2* affected the absorption and utilization of sulfate in *A. panax*.

**Figure 10:**
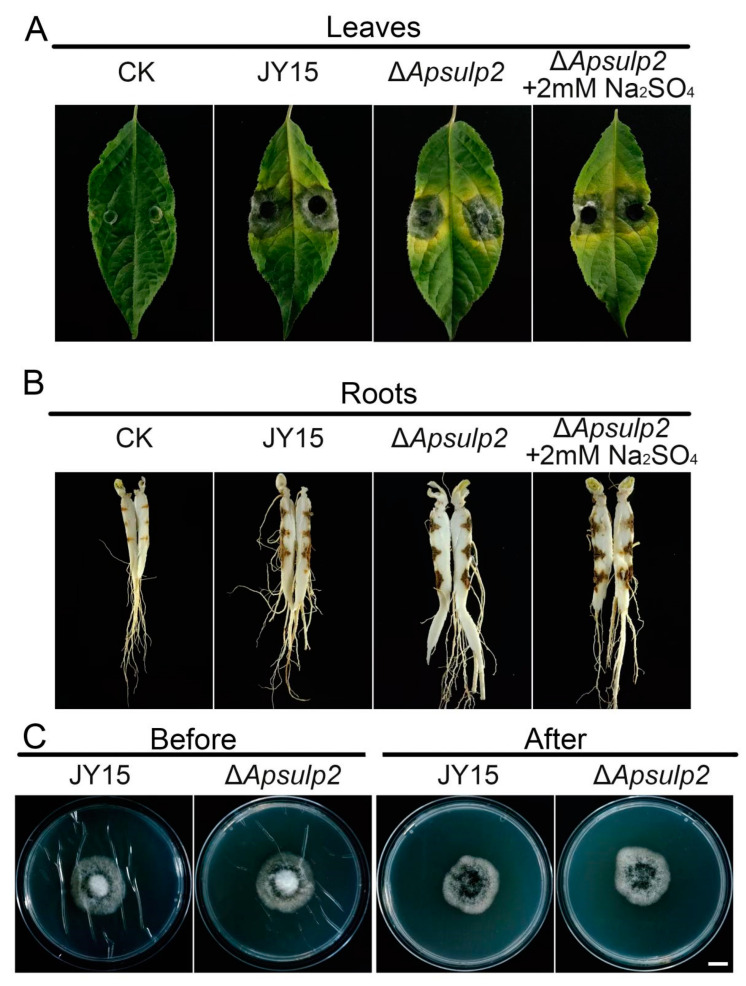
Pathogenicity assays of *A. panax* strains. (**A**,**B**) Pathogenicity analysis of the wild-type JY15 and Δ*Apsulp2* on ginseng leaves and roots. Agar plugs of the wild-type JY15 and Δ*Apsulp2* with grown mycelia were inoculated on detached ginseng leaves or roots and cultured in a moist chamber for 7 d. The ginseng roots were cut along their length and observed. PDA plugs were used as the control. (**C**) Penetration ability of the wild-type JY15 and Δ*Apsulp2* against a cellophane membrane. Three replicates were set up for each strain. Bar: 1 cm.

We also examined whether *ApSulP2* affects the pathogenicity against ginseng. The pathogenicity of the Δ*Apsulp2* mutant on ginseng leaves and roots did not differ from that of the wild-type JY15, with consistent disease symptoms observed (Figure 10A,B). Additionally, the results of a penetration ability assay indicate that the Δ*Apsulp2* mutant and the wild-type JY15 exhibited comparable cellophane membrane penetration capabilities (Figure 10C). These results imply that the Δ*Apsulp2* mutant did not influence the pathogenicity or penetration ability of *A. panax*, but it did impact the absorption and utilization of sulfate by *A. panax*.

## 4. Discussion

The WD40 family is a highly conserved superfamily of proteins that are widely present in the genomes of eukaryotic organisms. The WD40 proteins have been extensively characterized in higher eukaryotes. The WD40 domain proteins function as an adaptor in many different protein complexes or protein–DNA complexes in very diverse cellular processes [12]. They play diverse roles in processes such as growth and development, signal transduction, apoptosis, and immune responses [51,52,53]. In lower eukaryotes, such as filamentous fungi, the WD40 proteins are mainly involved in growth, development, and virulence. However, the biological functions exerted by the different WD40 repeat units also differ [54,55,56]. In this study, we characterized the role of a WD40 protein containing six tandem WD40 repeat structures. It was demonstrated that *ApWD40a* participates in multiple biological processes, including mycelial growth, conidiation, responses to various environmental stresses, secondary metabolite production, and pathogenicity in *A. panax*.

WD40 repeat domains containing proteins can participate in the vegetative growth and conidiation processes of plant pathogenic fungi [24,34,56]. In *V. dahliae*, the RACK1-like protein containing a WD40 repeat domain plays a key role in spore germination and morphogenesis [33]. Similarly, the disruption of the WD40 domain in *CreC* of *M. oryzae* and *M. robertsii* and *Tup1* of *F. oxysporum* was shown to significantly affect conidiation and formation [25,30]. In this study, the deletion of *ApWD40a* led to defects in the fungal growth and conidiation of *A. panax*. In general, although the WD40 repeat domains vary, they play a conserved role in fungal vegetative growth and conidiation in most plant pathogenic fungi.

Fungi frequently face environmental challenges, such as nutrient limitation, osmolarity, and exposure to toxic substances. The environmental stress response is essential for the fungus’s ability to survive stress. WD40 proteins are known to play a role in the stress response and carbon source utilization in filamentous fungi. In this study, the Δ*Apwd40a* mutant exhibited increased sensitivity to osmotic (sorbitol) and metal ion (NaCl and KCl) stress inducers (Figure 4), but decreased sensitivity to oxidative and cell wall stress factors, indicating the importance of *ApWD40a* in maintaining the *A. panax* stress response to the external environment and cell wall integrity (Figure 4). Notably, this differs from other genes encoding members of the WD40 protein family. For instance, in the protein coded by *CreC* of *M. oryzae* and *M. robertsii*, which contains WD40 repeat units, the inhibition rates increase in the presence of metal ions Na^+^ and K^+^, but in contrast to Δ*Apwd40a*, the Δ*creC* mutant also shows increased sensitivity to H_2_O_2_ and Congo red [25,30]. The decreased sensitivity to oxidative and cell wall stress factors in Δ*Apwd40a* suggests distinct roles for WD40 proteins in different filamentous fungi. Additionally, WD40 proteins were shown to act as important regulatory factors in carbon catabolite repression pathways. In *A. nidulans* and *M. oryzae*, as well as *M. robertsii*, the deletion of the *CreC* results in some carbon sources causing slight damage to *A. nidulans* [31], while in *M. oryzae* and *M. robertsii*, the mycelial growth rate decreases and utilization of various carbon sources is reduced [25,30]. Consistent with these findings, the Δ*Apwd40a* mutant in this study showed significantly reduced growth rates on different carbon sources, albeit to varying extents (Figure 3). These observations indicate that WD40 repeat units can affect the utilization of carbon sources.

The WD40-domain-containing proteins are involved in the virulence of phytopathogenic fungi; the disruption of genes such as *Rack1*, *MoTup1*, *FonTup1*, and *FonDoa1* was shown to reduce the pathogenicity of *U. maydis* [26], *M. oryzae* [24], and *F. oxysporum* [34,57]. Consistent with previous studies, our assessments of pathogenicity against ginseng leaves and roots revealed that the Δ*Apwd40a* mutant of *A. panax* exhibited decreased pathogenicity (Figure 5A,B). To explore the potential reasons for the reduced pathogenicity, we conducted penetration assays of the Δ*Apwd40a* mutant and found that the disruption of this gene did not affect the penetration ability of *A. panax* (Figure 5C). This result is in agreement with the findings for *FonTup1* in *F. oxysporum* but contrasts with the results for *BsTup1* in *Bipolaris sorokiniana* [34,58].

Most necrotrophic fungi initially kill their host plants’ cells and then absorb nutrients from the dead cells to accomplish the invasion of the host plant. During this process, plant pathogenic fungi secrete a series of unique and complex secondary metabolites, such as toxins and melanin, which are often important for the pathogenicity of the fungi. To date, it has been reported that *Alternaria* spp. can produce over 300 secondary metabolites [59]. A host-specific ACT toxin in the acetic acid ethyl ester extracts of the culture filtrates and spore germination fluid of *A. alternata* plays a significant role in pathogenesis. It was demonstrated that the transcription factors *StuA*, *Tfb5*, *ACTTS2*, *ACTR*, and *pacC* all regulate the biosynthesis of the ACT toxin [60,61,62,63]. Most recently, in 2024, the targeted isolation of mycotoxins was performed in *A. panax* and tyrosol was identified to have the highest toxicity among the various isolated small-molecule compounds [9]. In this study, we found that the content of tyrosol in Δ*Apwd40a* was significantly reduced. Additionally, another secondary metabolite, melanin, can protect the ability of fungi to invade hosts and resist environmental stress [64]. Previously, the deletion of melanin-synthesis-related genes *ALB1*, *BUF1*, and *RSY1* resulted in Δ*alb1*, Δ*buf1*, and Δ*rsy1* mutants that did not cause any lesions, confirming that melanin and its synthetic genes are necessary for the virulence of the wild-type strains Guy11 and 70–15 of *M. oryzae* [48]. The results of the present study confirm that the deletion of the *ApWD40a* leads to a reduction in melanin synthesis. It can be speculated that *ApWD40a* regulates the synthesis of secondary metabolites, such as melanin and toxins, and thereby potentially decreases the pathogenicity of *A. panax*.

Transcriptome analysis between the Δ*Apwd40a* mutant and the wild-type JY15 showed the possible role of *ApWD40a* in *A. panax*. In this study, 832 DEGs were identified in the Δ*Apwd40a* mutant (Figure 7A), similar to the number of DEGs in *FonTup1*, with more upregulated than downregulated genes [34]. The upregulated DEGs were notably involved in various metabolic pathways encompassing a range of amino acid metabolisms and glutathione metabolism (Figure S5). Sulfate transport plays a crucial role in various pathways, including the biosynthesis of sulfate-containing amino acids and cysteine and the metabolism of glutathione. We found that in *A. panax*, a gene (G6011_04785) that encoded sulfate permease 2, named *ApSulP2,* was significantly upregulated in the Δ*Apwd40a* mutant (Table S5). It has been reported in *A. nidulans* that the WD40 domain is associated with the regulation of sulfur metabolism, and the inhibitory effect on sulfur metabolic products is relieved in knockout mutants [22]. In this study, we found that *ApSulP2* appeared to be involved in the utilization of sulfate, as the mycelial growth of the Δ*Apsulp2* mutant was sparse, with a significant decrease in the biomass, on the media that lacked or contained low levels of sulfate (Figure 9). Similarly, it was reported for *A. nidulans* that the *sB1*^pr^ mutant (M80) cannot grow on MM with sulfate as the sole sulfate source, whereas subsequent strains obtained by crossing that strain with the M80 strain carrying the *sB1*^pr^ mutation (strains M81 to M90) grew quite well in liquid MM supplemented with 2 mM sulfate [65]. Likewise, similar to *A. nidulans*, the exogenous addition of sulfate resulted in an increase in the biomass of the Δ*Apsulp2* mutant herein. The disruption of *ApSulP2* did not affect the penetration ability of the pathogen or influence the pathogenicity of *A. panax*. Our study for the first time indicates the importance of *ApSulP2* in the basic biological processes of plant pathogenic fungi. The results imply that *ApWD40a* regulates the expression of *ApSulP2* to modulate sulfate assimilation and metabolism in *A. panax*.

## Data Availability

The original contributions presented in this study are included in the article/Supplementary Materials. Further inquiries can be directed to the corresponding authors.

## Supplementary Materials

The following supporting information can be downloaded at: https://www.mdpi.com/article/10.3390/jof11010059/s1, Figure S1: Amino acid sequence alignments of ApWD40a with homologs from different fungal species; Figure S2: Knockout and complement of the *ApWD40a* gene; Figure S3: Proportion of the sequencing reads mapped on *Alternaria panax* genome and correlation analysis; Figure S4: Enrichment of GO terms for the upregulated and downregulated DEGs in Δ*Apwd40a*; Figure S5: Enrichment of KEGG terms for the upregulated and downregulated DEGs in Δ*Apwd40a*; Figure S6: Sequence alignment of ApSulp2 with *Aspergillus nidulans* Sulp2 proteins; Figure S7: Knockout of the *ApSulp2* gene; Table S1: Primers used in this study; Table S2: Data quality control and statistics of RNA-Seq reads mapped on *Alternaria panax* genome; Table S3: Enrichment of GO terms of the differentially expressed genes in Δ*Apwd40a*; Table S4: Enrichment of KEGG pathways of the differentially expressed genes in Δ*Apwd40a*; Table S5: Expression of the *ApSulp2* gene in the Δ*Apwd40a*.
