# Supplementary material for: ApWD40a, a Member of the WD40-Repeat Protein Family, Is Crucial for Fungal Development, Toxin Synthesis, and Pathogenicity in the Ginseng Alternaria Leaf Blight Fungus Alternaria panax
Source: J Fungi (Basel). 2025 Jan 14;11(1):59. doi: 10.3390/jof11010059 (PMC11767187; doi:10.3390/jof11010059)
Supplement: Supplementary file 1 [file jof-11-00059-s001.zip › jof-3385838-supplementary.pdf]

Supporting Information

ApWD40a, a Member of the WD40-Repeat Protein Family, Is Crucial for Fungal Development, Toxin Synthesis, and Pathogenicity in the Ginseng Alternaria Leaf Blight Fungus *Alternaria panax*

Jinling Lan [1], Shengjie Mei [1], Yingxue Du [1], Meili Chi [2], Jiayi Yang [1], Shuliu Guo [1], Mingliang Chu [1], Ronglin He [1,*] and Jie Gao [1,*]

[1] College of Plant Protection, Jilin Agricultural University, Changchun 130118, China; jinling_lan@aliyun.com (J.L.)

[2] National Ginseng Products Quality Inspection Testing Center, Yanji 133000, China; 15944330016@163.com

* Correspondence: hrl1007@163.com (R.H.); jiegao115@126.com (J.G.)

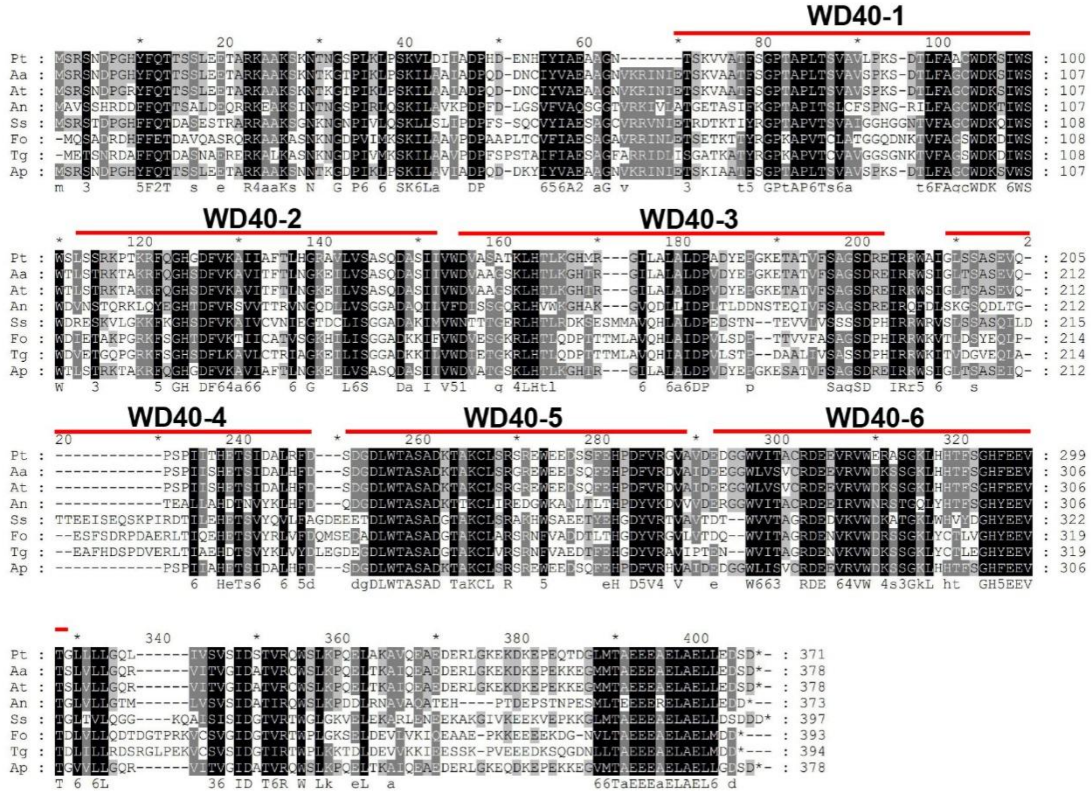

Figure S1. Amino acid sequence alignments of ApWD40a with homologs from different fungal species. Amino acid sequences of the WD40a proteins used are as follows: Pt: *Pyrenophora teres* EFQ89095.1; Aa: *Alternaria alternata* KAH6846796.1; At: *Alternaria tenuissima* RYN38094.1; An: *Aspergillus nidulans* XP_658660.1; Ss: *Sclerotinia sclerotiorum* XP_001597417.1; Fo: *Fusarium oxysporum* EGU75440.1; Tg: *Trichoderma gamsii* XP_018663274.1; Ap: *Alternaria panax* JY15.

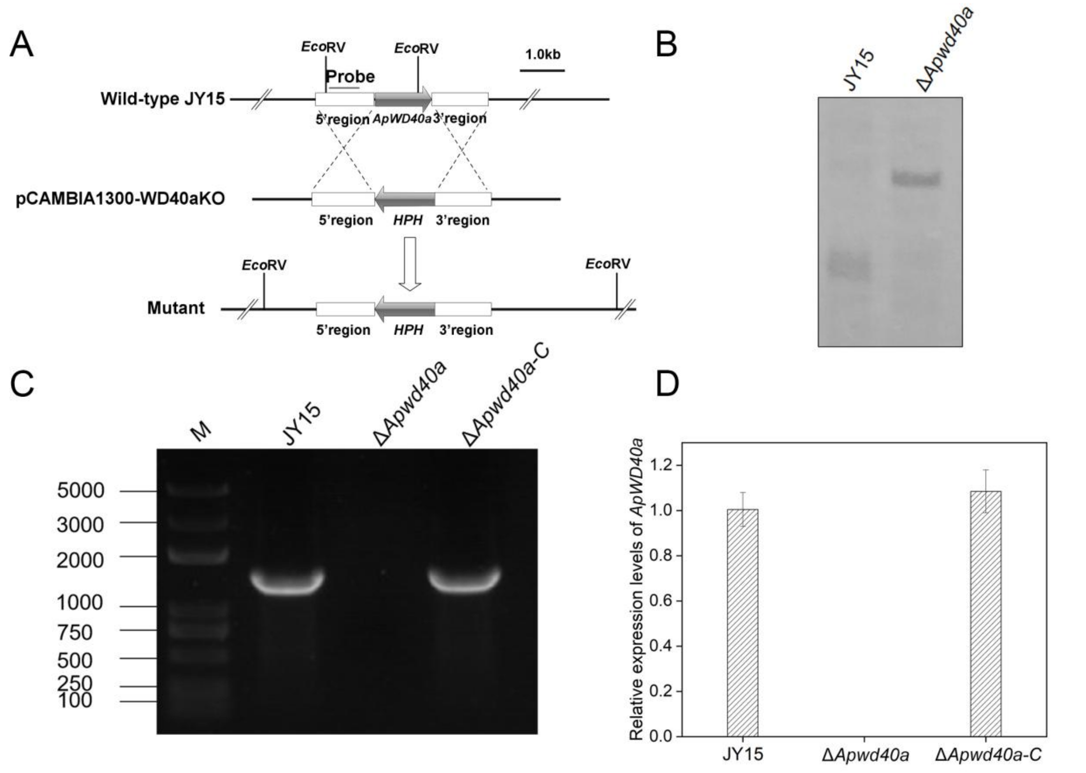

Figure S2. Knockout and complement of the *ApWD40a* gene. A: Strategy of *ApWD40a* gene knockout; B: Southern blot analysis of the wild-type JY15 and the null mutant; C: PCR validation of complement transformants; D: Relative expression of the complement mutants Δ*Apwd40a-C* determined by qRT-PCR.

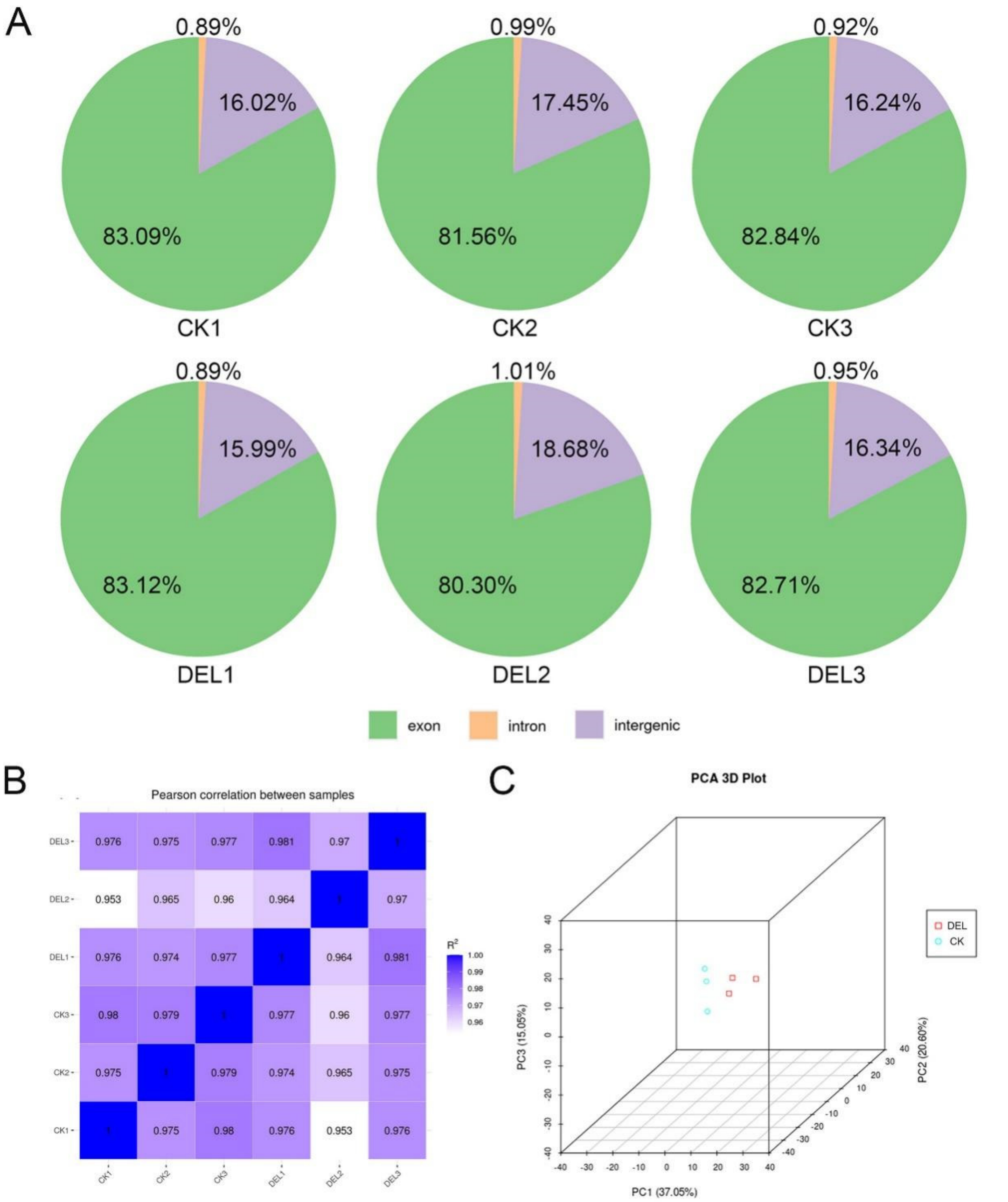

Figure S3. Proportion of the sequencing reads mapped on *Alternaria panax* genome (A) and correlation analysis (B-C)

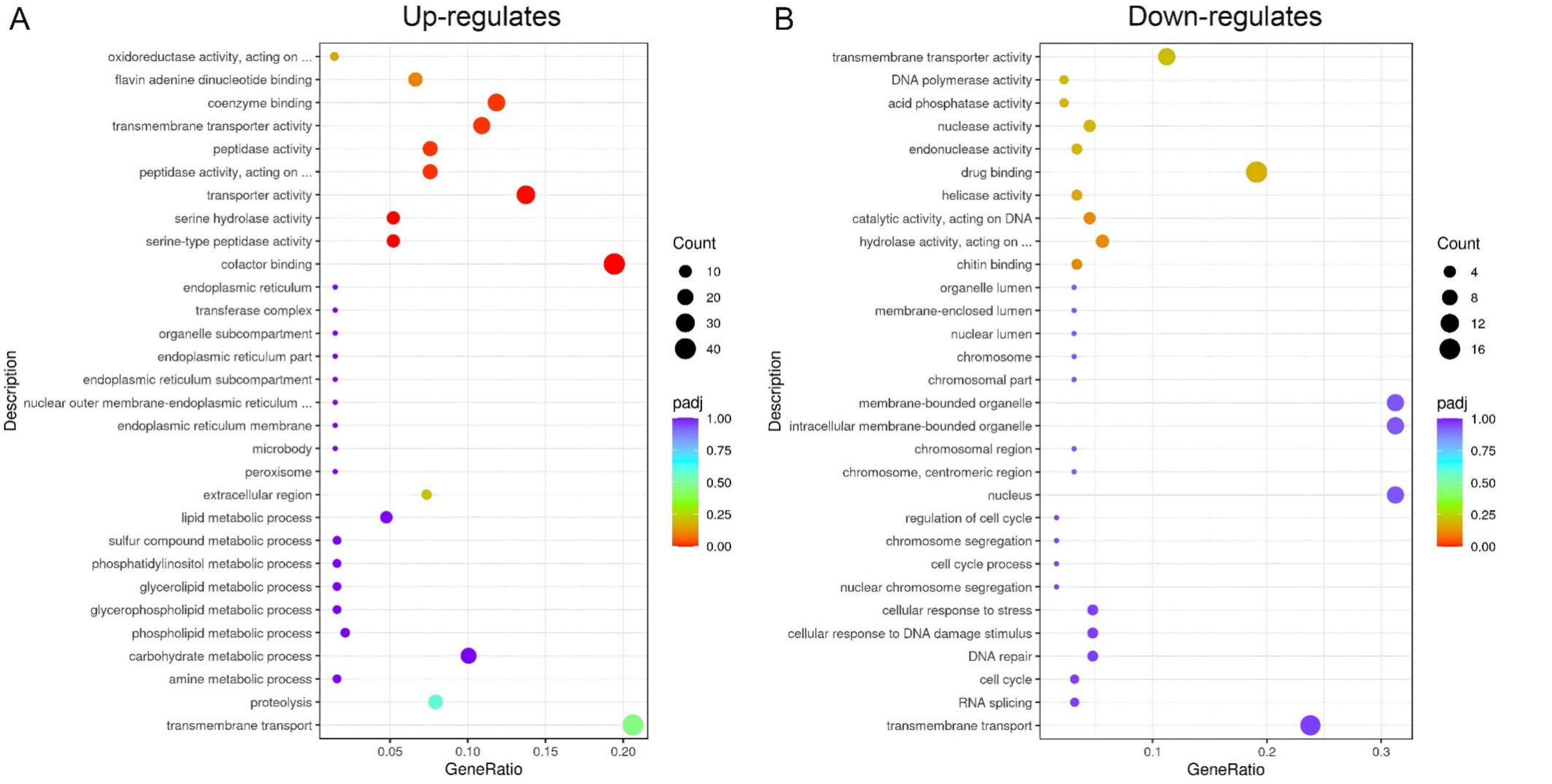

Figure S4. Enrichment of GO terms for the upregulated (A) and downregulated (B) DEGs in Δ*Apwd40a*. The size of each circle represents the number of genes number involved in the corresponding pathway.

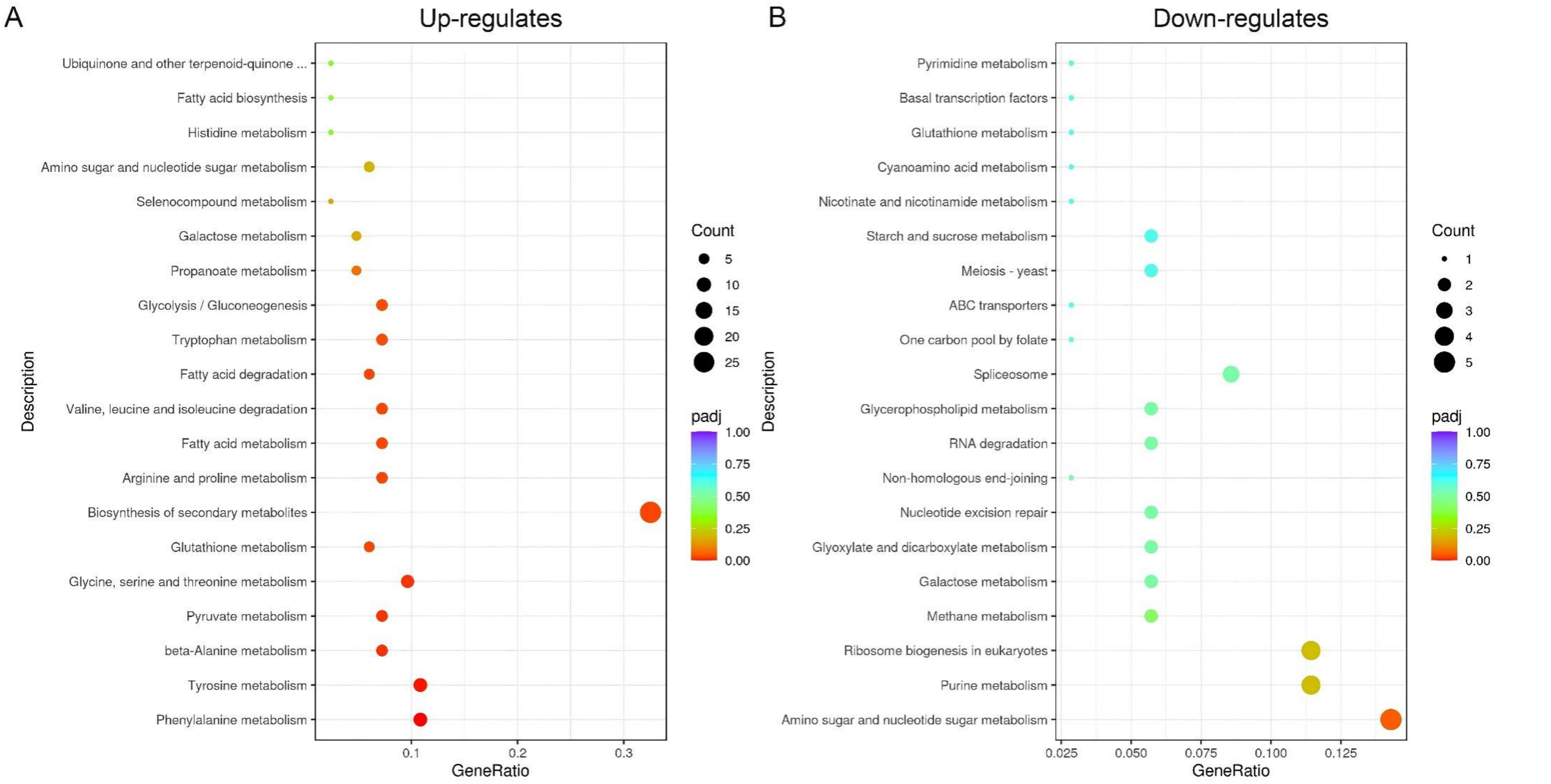

Figure S5. Enrichment of KEGG terms for the upregulated (A) and downregulated (B) DEGs in Δ*Apwd40a*. The size of each circle represents the number of genes number involved in the corresponding pathway.

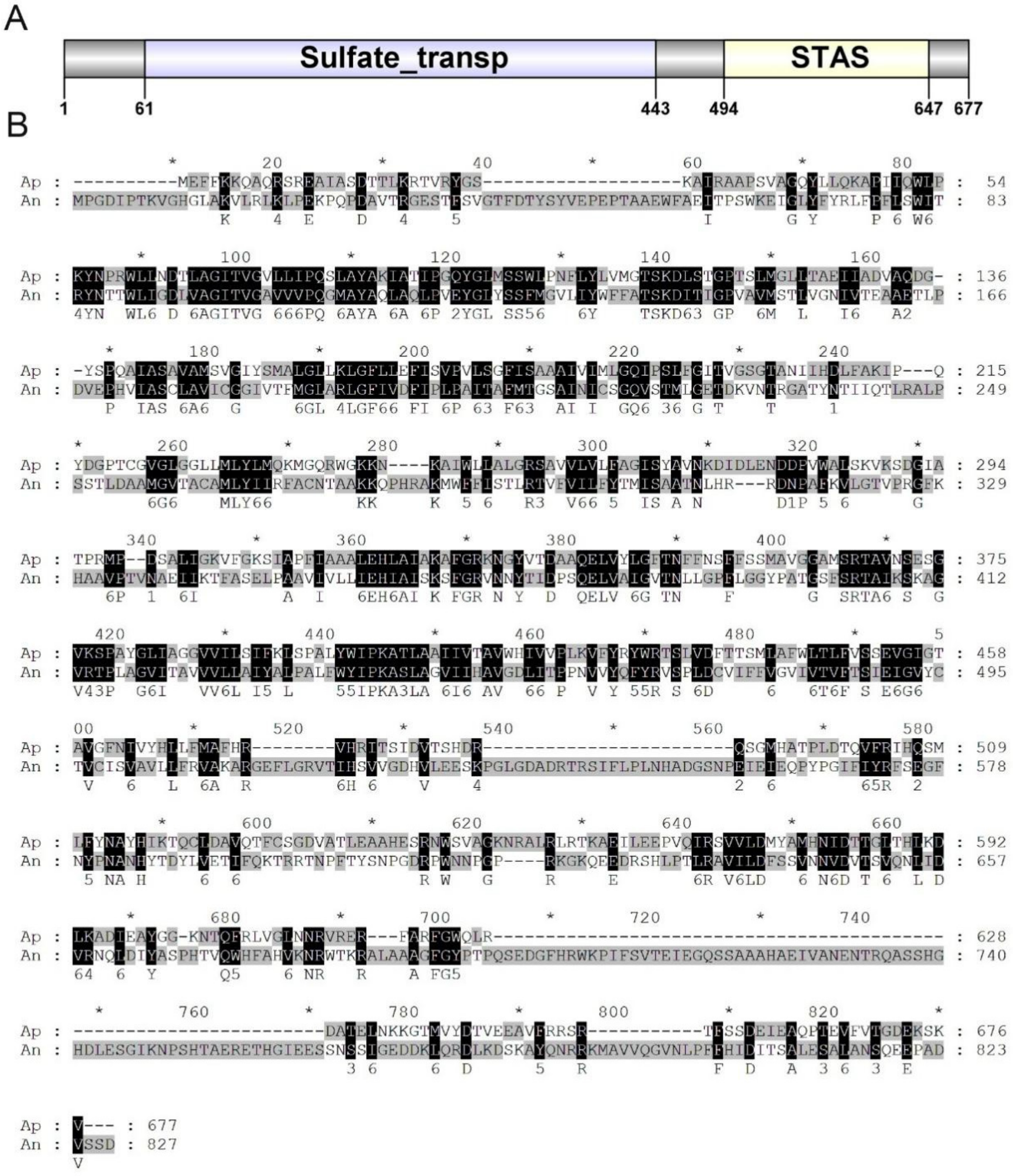

Figure S6. Sequence alignment of ApSulp2 with *Aspergillus nidulans* Sulp2 proteins. The Sulp2 protein sequence used is as follows: *Aspergillus nidulans* FGSC A4 AnSulp2 (XP_660334.1).

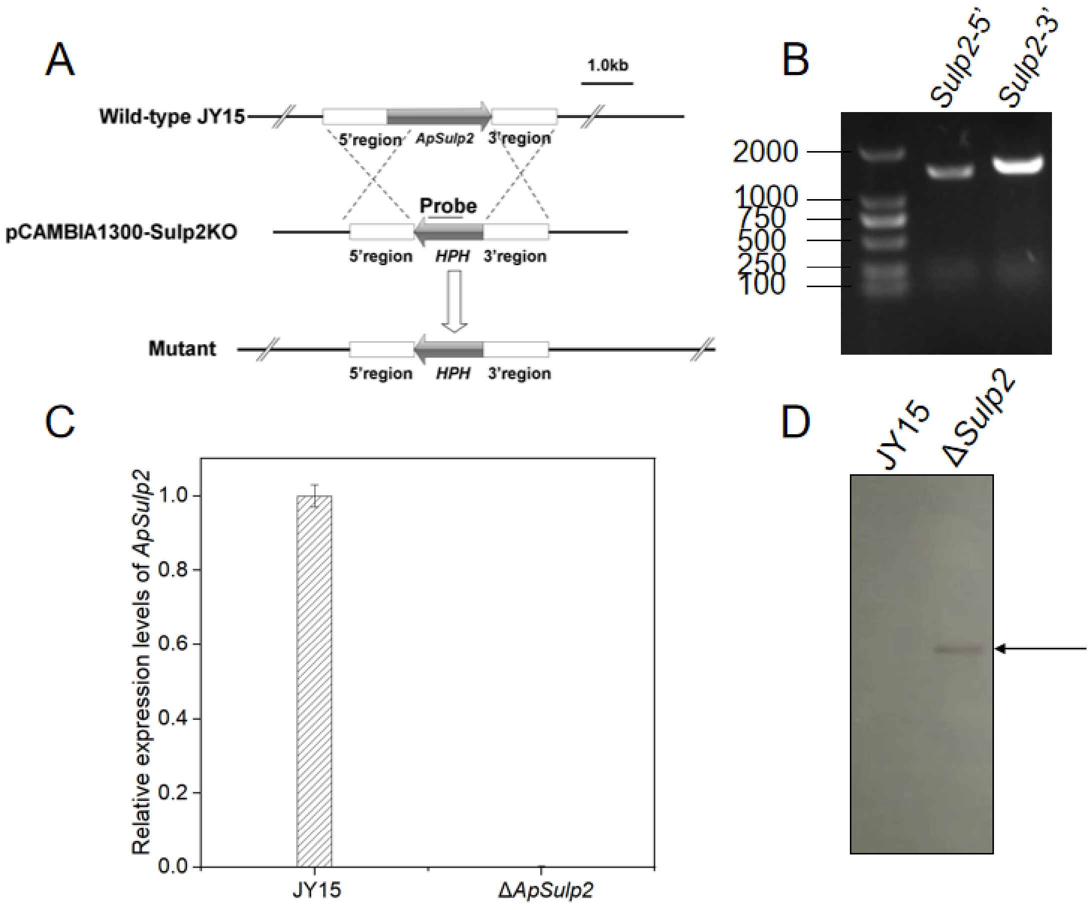

Figure S7. Knockout of the *ApSulp2* gene. A: Strategy of *ApSulp2* gene knockout; B: 5′ and 3′ flanking PCR validation of knockout transformants; C: Relative expression level of the target gene *ApSulp2* in the wild-type JY15 and the Δ*Apsulp2* mutant; D : Single copy analysis of the *Hyg* gene in the null mutant of *ApSulp2*.

Table S1. Primers used in this study

Primers	Sequences (5'- 3')
**Construction and verification of deletion mutants**	
WD40a-5F	CGAGCTCGGTACCCGGggatccCTTCGCCGCCGCCAGTG
WD40a-5R	GATGTGTTGACCTCCCGGTGACGCCCAGCAAGATA
WD40a-3F	AAAGGAATAGAGTAGTCTTCGACGAGCGGACCACTACC
WD40a-3R	ACGACGGCCAGTGCCaagcttGCCCAGCCATCAACGAGGAAACTA
WD40a-Hyg-F	TGCTGGGCGTCACCGGGAGGTCAACACATCAATGCT
WD40a-Hyg-R	TCCGCTCGTCGAAGACTACTCTATTCCTTTGCCCTC
Sulp2-5F	CGAGCTCGGTACCCGGggatccTCCAAGTACCCGCTTCATAGC
Sulp2-5R	GATGTGTTGACCTCCTCAACCGCAAGATAGCCTCAT
Sulp2-3F	AAAGGAATAGAGTAGGCCGTCTGCTCTGCGACACT
Sulp2-3R	ACGACGGCCAGTGCCaagcttCCTCCGAAGAATTAAGTCCAGTT
Sulp2-Hyg-F	AGGCTATCTTGCGGTTGATAGGAGGTCAACACATCAATGCT
Sulp2-Hyg-R	GTCGCAGAGCAGACGGCCTACTCTATTCCTTTGCCCTC
1300F1	ATTCGAGCTCGGTACCCGGG
*Hyg*R1	CCAAAATAGCATTGATGTGTTGAC
*Hyg*F2	CGAGGGCAAAGGAATAGAGT
1300R2	CTAATAAACGCTCTTTTCTCTTA
RPB2-F	TGTCACAGTTGTGCAGGAGCT
RPB2-R	TTTCGCAATGGGTGTAGGC
WD40a-F	GTCAAGGCCGTCATCGCATTCAC
WD40a-R	GCCATCCGCCGTCTTCGTCA
WD40a-upF	ATCCATGCCAACCAAACCCACCAA
WD40a-upR	TGCACCAAGCAGCAGATGA
WD40a-downF	TCCGAGGGCAAAGGAATAGA
WD40a-downR	CCCCCTTGTAGCTCGGTCTGG
Sulp2-F	TGGTGGTGTCGTCATCCTCA
Sulp2-R	CGCTCCAGTTACGGCTTTCG
Sulp2-upF	AACCAAGTTGTCGCATCCAC
Sulp2-upR	GTCCTCGTTCCTGTCTGCTAAT
Sulp2-downF	ACTCGCCGATAGTGGAAACC
Sulp2-downR	GTGGACCGCTCGGAGACAT
Hyg-probe-F	GGAGGTCAACACATCAATG
Hyg-probe-R	CTACTCTATTCCTTTGCCCTCG
WD40a-probe-F	TGTTATGATGGGCGGTGCG
WD40a-probe-R	TAGGGTGGGAGGAGCGGTGA
**Construction and verification of complement mutants**	
WD40a-ProF	GTACCCGGGGATCCTtctagaCAAACCCTGACGACCCAAAT
WD40a-ProR	GTTTGAGCGAGACATGGTGACGCCCAGCAAGATA
HB*WD40a*-F	TGCTGGGCGTCACCATGTCTCGCTCAAACGATCCA
HB*WD40a-R*	TCCGCTCGTCGAAGAATCGCTATCTCCCAGAAGCTC
WD40a-TerF	CTGGGAGATAGCGATTCTTCGACGAGCGGACCACTA
WD40a-TerR	ACGACGGCCAGTGCCaagcttGAATGAAGCCAGGAATCTGT
**RT-qPCR assays**	
q*Hyg*-F	GCGTTTGTCAAGCAAGGTAAG
q*Hyg*-R	GATCGAGGCTTGGGTAGAATAG
q*RPB2*-F	TTCACTCCAACGCTCAACAG
q*RPB2*-R	GGTCACGAATGTCACGAATCA

q*WD40a*-F	CAAACGATCCAGGTCACTACTT
q*WD40a*-R	CCTTGGTGTTGTTGGACTTTG
q*Sulp2*-F	TGGCTACCAAACTTTCTCTACC
q*Sulp2*-R	CCTGAGCAACATCTGCAATTATC
qG6011_00826F[a]	GAATTTCCGGCCTTGAATGTATC
qG6011_00826R	GACGATGTTGGGATGAGATAGG
qG6011_01906F	CTGATCCCACATGGTCCATAAC
qG6011_01906R	CCGTCAAGACAGTCGCAATAA
qG6011_10931F	CACGTCCGACGAGAAGTATTG
qG6011_10931R	CCCTCCACACGTAGTTTGTT
qG6011_02567F	TGCCACCTACAAGAACAATGA
qG6011_02567R	GAGAGATGAGATGGTTGGGATG
qG6011_02791F	CGCTTACCGCATACCTACTATC
qG6011_02791R	CATCAACCCATTGCCGTATTT
qG6011_03601F	CACACACTCTGGGATGACTTT
qG6011_03601R	TACCAACGCGTGGTGATATG
qG6011_03829F	CATGTCCGACGAAGAACTTACT
qG6011_03829R	GTGTGAGATACGACCCAATCC
qG6011_04126F	TCCCTCGCCATGGTTTATTC
qG6011_04126R	CGACAGCTTGTAGTGCTTCT
qG6011_04138F	ATCTCCGACCACCCTACAA
qG6011_04138R	TACTCTCGAGCTTCGCAATTC
qG6011_04402F	GGATGCTACCTACAACGTCTAC
qG6011_04402R	CTCACTAGCTTTGGGAGAGAAG
qG6011_04785F	TGGCTACCAAACTTTCTCTACC
qG6011_04785R	CCTGAGCAACATCTGCAATTATC
qG6011_07253F	CCAGCTCATGTACGATGTCA
qG6011_07253R	GGTATGGTCGTCGAAAGAGTAG
qG6011_07538F	TTGCAGCCTATGTCGTTCTC
qG6011_07538R	CCGGACTTCAGGCATATCTTT
qG6011_08141F	CATACTTCCAGATGCCGACTAC
qG6011_08141R	GTCCTGTTCCCATAGCTGATAC
qG6011_09959F	CGAACGACAGCGTGGATAA
qG6011_09959R	CTCGGTGTCAGTCCAAGAAA

[a]Notes:

G6011_00826, GenBank accession number: KAG9195705.1, gene name: *NEK2*;

G6011_01906, GenBank accession number: KAG9187983.1, gene name: *GalE*;

G6011_10931, GenBank accession number: KAG9192197.1, gene name: *EIF2α*;

G6011_02567, GenBank accession number: KAG9186011.1, hypothetical protein;

G6011_02791, GenBank accession number:KAG9186235.1, gene name: *IDO1*;

G6011_03601, GenBank accession number:KAG9193566.1, gene name: *TYRP1*;

G6011_03829, GenBank accession number:KAG9193794.1, gene name: *Pr1*;

G6011_04126, GenBank accession number:KAG9194091.1, gene name: *PTR2*;

G6011_04138, GenBank accession number:KAG9194103.1, hypothetical protein;

G6011_04402, GenBank accession number:KAG9194367.1, gene name: *MEP2*;

G6011_04785, GenBank accession number:KAG9194750.1, gene name: *SulP2*;

G6011_07253, GenBank accession number:KAG9185922.1, hypothetical protein;

G6011_07538, GenBank accession number:KAG9188833.1, gene name: *TRI101*;

G6011_08141, GenBank accession number:KAG9190053.1, gene name: *DHX16*;
G6011_09959, GenBank accession number:KAG9186851.1, gene name: *Pol4.*

Table S2. Data quality control (Table S2A) and statistics of RNA-Seq reads mapped on *Alternaria panax* genome (Table S2B)

A.

Sample_ Name	Clean_ reads	Clean_ bases	Error_ rate	Q20	Q30	CG
CK1	42747474	6.41G	0.03	97%	91.88%	56%
CK2	45209334	6.78G	0.03	96.76%	92.45%	55.7%
CK3	43375358	6.51G	0.03	97.66%	93.79%	56%
DEL1	44805016	6.72G	0.03	97.3%	92.79%	55.87%
DEL2	41996816	6.3G	0.03	97.32%	92.92%	55.63%
DEL3	44268042	6.64G	0.03	97.18%	92.52%	55.94%

B.

Sample	Total_reads	Total_map	Unique_map	Multiple_map
CK1	42747474	41120074(96.19%)	40446835(94.62%)	673239(1.57%)
CK2	45209334	43139665(95.42%)	42368714(93.72%)	770951(1.71%)
CK3	43375358	41623240(95.96%)	40832566(94.14%)	790674(1.82%)
DEL1	44805016	43032559(96.04%)	42264560(94.33%)	767999(1.71%)
DEL2	41996816	40235439(95.81%)	39280314(93.53%)	955125(2.27%)
DEL3	44268042	42583370(96.19%)	41902141(94.66%)	681229(1.54%)

Table S3. Enrichment of GO terms of the differentially expressed genes in Δ*Apwd40a*

Category	GOID	Description	padj
BP	GO:0055085	transmembrane transport	0.032000948
MF	GO:0005215	transporter activity	0.001211536
MF	GO:0048037	cofactor binding	0.001211536
MF	GO:0022857	transmembrane transporter activity	0.003186695
MF	GO:0008236	serine-type peptidase activity	0.007926748
MF	GO:0017171	serine hydrolase activity	0.007926748
MF	GO:0050662	coenzyme binding	0.032838847

Note: padj represents the corrected *p*-value. The padj value < 0.05 means a most enriched GO term.

BP represent biological process, MF represent molecular function.

Table S4. Enrichment of KEGG pathways of the differentially expressed genes in Δ*Apwd40a*

KEGG ID	Description	GeneRatio	BgRatio	*p*-value	padj
aalt00360	Phenylalanine metabolism	9/118	35/2252	4.89E-05	0.003375103
aalt00350	Tyrosine metabolism	10/118	56/2252	0.000488644	0.013034596
aalt00520	Amino sugar and nucleotide sugar metabolism	10/118	57/2252	0.000566722	0.013034596
aalt00480	Glutathione metabolism	6/118	29/2252	0.003206746	0.052173861
aalt00260	Glycine, serine and threonine metabolism	9/118	61/2252	0.003780715	0.052173861
aalt00410	beta-Alanine metabolism	6/118	31/2252	0.004560904	0.052450399
aalt00620	Pyruvate metabolism	6/118	35/2252	0.008469627	0.083486325
aalt00052	Galactose metabolism	6/118	38/2252	0.012671	0.109287379
aalt00330	Arginine and proline metabolism	6/118	43/2252	0.02261348	0.157414778
aalt00071	Fatty acid degradation	5/118	32/2252	0.023431276	0.157414778
aalt01212	Fatty acid metabolism	6/118	44/2252	0.02509511	0.157414778
aalt00280	Valine, leucine and isoleucine degradation	6/118	45/2252	0.027752879	0.159579054
aalt00680	Methane metabolism	4/118	24/2252	0.033730313	0.179030121
aalt00380	Tryptophan metabolism	6/118	48/2252	0.036824591	0.181492628
aalt00010	Glycolysis / Gluconeogenesis	6/118	49/2252	0.040226548	0.185042119
aalt01110	Biosynthesis of secondary metabolites	31/118	446/2252	0.048631299	0.209722476

Table S5. Expression of the *ApSulp2* gene in the Δ*Apwd40a*

Description	Gene_ID	log_2FoldChange	padj
Sulp2	G6011_04785	2.212936241	0.000268382
